# Isotopic labelling studies for a gold-catalysed skeletal rearrangement of alkynyl aziridines

**DOI:** 10.3762/bjoc.7.96

**Published:** 2011-06-21

**Authors:** Paul W Davies, Nicolas Martin, Neil Spencer

**Affiliations:** 1 School of Chemistry, University of Birmingham, Edgbaston, Birmingham, B15 2TT, United Kingdom

**Keywords:** 1,2-aryl shift, cycloisomerisation, gold, isotopic labelling, pyrrole, skeletal rearrangement

## Abstract

Isotopic labelling studies were performed to probe a proposed 1,2-aryl shift in the gold-catalysed cycloisomerisation of alkynyl aziridines into 2,4-disubstituted pyrroles. Two isotopomers of the expected skeletal rearrangement product were identified using ^13^C-labelling and led to a revised mechanism featuring two distinct skeletal rearrangements. The mechanistic proposal has been rationalised against the reaction of a range of ^13^C- and deuterium-labelled substrates.

## Introduction

Gold-catalysed cycloisomerisation reactions have emerged as powerful methods to construct a diverse array of hetero- and carbocyclic motifs under generally mild reaction conditions using straightforward preparative procedures [1–9]. Many such processes also incorporate a rearrangement of the hydrocarbon skeleton present in the starting material, which can lead to unexpected and synthetically interesting outcomes [10–11].

One of the most common fundamental steps in these skeletal rearrangements involves C–C bond fission through 1,2-migration. This step is triggered by the generation of carbocationic character, which is generally stabilised in some form, either by an adjacent gold atom or through extended delocalisation. While 1,2-alkyl migrations are well-established in gold-catalysed heterocyclic synthesis [12–13], 1,2-aryl shifts are much less common [14–17].

**Scheme 1:**
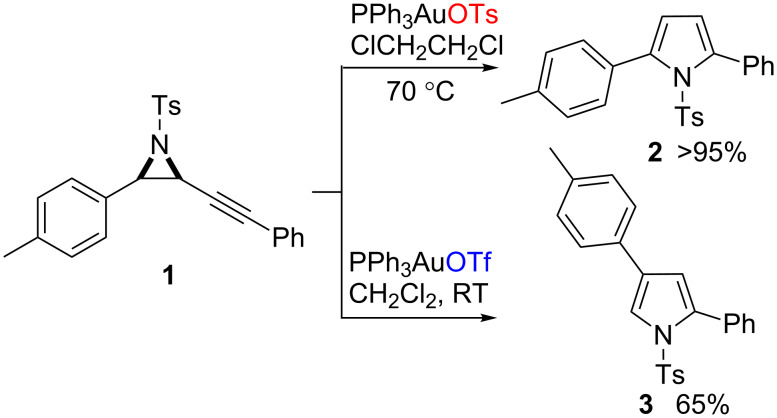
Gold-catalysed cycloisomerisations of aryl–alkynyl aziridine to pyrroles.

In relation to these processes, we recently reported the gold-catalysed reactions of aryl substituted alkynyl aziridines, such as **1**. Careful choice of reaction conditions resulted in selective cycloisomerisation to produce either the 2,5-disubstituted pyrrole **2** or the skeletally rearranged 2,4-disubstituted pyrrole **3** (Scheme 1) [18].

The nature of both the solvent, and the counter ion to the cationic gold catalyst, proved crucial to the reaction outcome: 2,4-Disubstituted pyrroles were only observed in appreciable quantities when the reaction was performed with a non basic solvent and counter ion combination that allows for formation of a separated ion pair, such as dichloromethane and triflate (Scheme 1). This selectivity was switched and the 2,5-disubstituted products could be achieved in quantitative yields when the counter ion was the more basic tosylate [19]. Alternatively, the use of protic, ethereal or aromatic solvents favoured the formation of the 2,5-disubstituted products regardless of the counter ion. Similar solvent regimes were employed in other reports into π-acid promoted alkynyl aziridine cycloisomerisations without skeletal rearrangement [20–23].

**Scheme 2:**
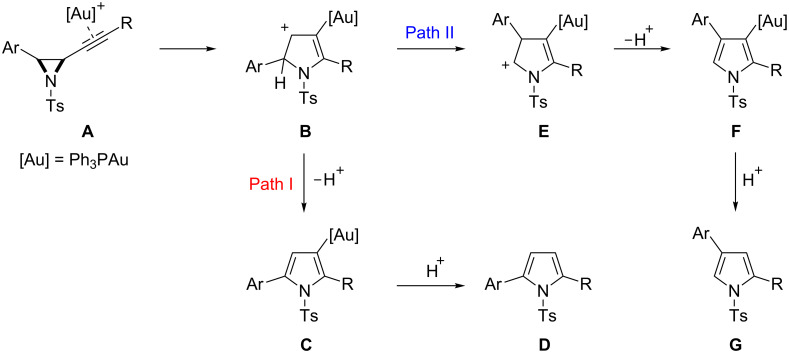
Working mechanism to rationalise the formation of two regiosomeric pyrroles in the gold catalysed cycloisomerisation of alkynyl aziridines.

Our working mechanism to explain this reaction divergence centred on the electrophilic activation of the alkyne in **A** triggering a ring-expansion to a common intermediate **B** from which both isomeric products **D** and **G** evolved (Scheme 2). Path I follows proton elimination (**B**→**C**) and subsequent protodemetallation to give 2,5-disubstituted pyrrole **D**, in keeping with a previously reported furan synthesis [24–25]. The 2,4-disubstituted skeletal rearrangement product **G** was rationalised through Path II, where a 1,2-aryl shift (**B**→**E**) precedes proton elimination (**E**→**F**) and protodemetallation. The observed solvent and counter ion effects could be rationalised by considering that the 1,2-aryl shift at **B** is disfavoured relative to proton elimination as its cationic character is diminished, either by solvent stabilisation or the formation of a closer contact ion pair, and/or by more basic solvents/counter ions assisting proton elimination [18,26–27].

However, for the proposed skeletal rearrangement mechanism to be valid, gold bound carbocation **B** must be prone to undergo a relatively rare 1,2-aryl shift in preference to either proton elimination or a 1,2-hydride shift. We therefore sought to validate this proposal experimentally by isotopic labelling.

## Results and Discussion

### A deuterium labelled precursor

**Scheme 3:**
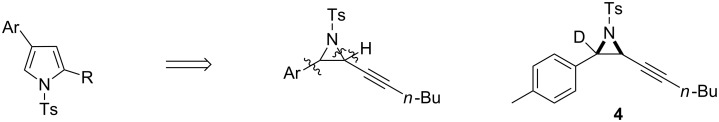
Bond fissions featured in the proposed mechanistic hypothesis and the initial mechanism probe.

Alkynyl aziridine **4** was selected as the initial probe for the mechanism which requires fission of three bonds: The propargylic C–N bond in ring expansion; the aryl–aziridinyl C–C bond in the 1,2 shift and the propargylic C–H bond for aromatisation (Scheme 3). The positioning of a deuterium atom at the benzylic carbon in **4** enables the carbons of the aziridine ring to be distinguished and labels the carbon from which the aryl shift would occur. The tolyl and butyl groups were selected as substituents in place of other aromatic units to aid analysis by simplifying the key aromatic region in the resulting NMR spectra.

**Scheme 4:**
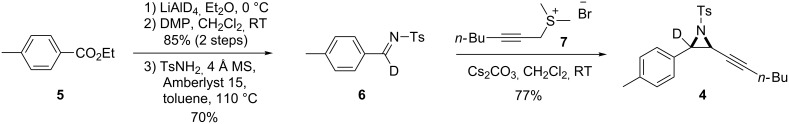
Preparation of D-labelled alkynyl aziridine **4**. DMP = Dess–Martin periodinane.

Deuterated aziridine **4** was prepared in four steps from ester **5** (Scheme 4). Reduction using lithium aluminium deuteride led to the insertion of the label at the benzylic position. Oxidation to the aldehyde followed by condensation with tosylamide afforded the deuterated imine **6**. Aziridination using the sulfonium ylide generated in situ from **7** proceeded smoothly to afford the desired cycloisomerisation precursor **4** [28]. The aziridines were formed as a mixture of diastereomers, with the *cis* diastereomer predominating, and were employed as such in the cycloisomerisation reactions.

**Scheme 5:**
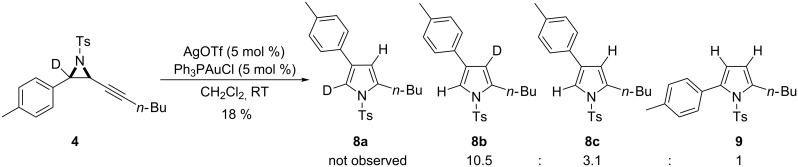
Reaction of deuterated alkynyl aziridine **4** in the skeletal rearrangement reaction.

Deuterated aziridine **4** was reacted under the standard conditions for skeletal rearrangement (5 mol % of Ph_3_PAuOTf generated in situ in CH_2_Cl_2_), to give a mixture of 2,4- and 2,5-pyrrole isomers **8c** and **9** alongside the main product, 3-deutero-2,4-disubstituted pyrrole **8b** (Scheme 5). Isomer **8a**, which was predicted as the major product under the proposed mechanism (Scheme 2), could not be indentified from the reaction mixture. Though H/D exchange in pyrroles has been noted in gold-catalysed reactions [13], control reactions had shown that no H/D exchange occurred in this specific system [18–19]. There was also no interconversion between 2,4- and 2,5-isomers under the same reaction conditions [18]. Surprisingly, despite several repetitions and the use of different batches of starting materials, the reaction of **4** was always less clean than its non-deuterated analogue, even when the substrates were tested side-by-side under identical conditions.

Despite this unexplained difference, the proposed mechanism does not account for the formation of 2,4-disubstituted pyrrole without deuterium incorporation at the C-5 position. More information on whether a 1,2-aryl shift occurs was therefore sought using a non-labile ^13^C label at the aziridine carbon.

### ^13^C labelling studies

**Scheme 6:**
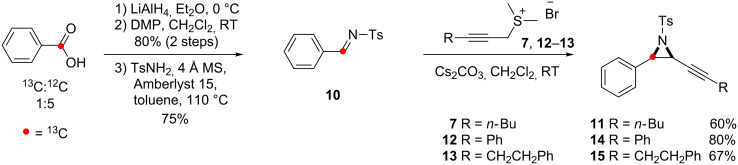
Preparation of ^13^C-enriched alkynyl aziridines.

^13^C-Enriched benzoic acid was converted into imine **10** and subsequently coupled with **7** to give the aziridine **11**, in four steps, as a 1:5 ^13^C:^12^C mixture (Scheme 6). Two further ^13^C-enriched alkynyl aziridines, **14** and **15**, with different substituents on the alkyne, were later prepared using the sulfonium salts **12** and **13**.

**Scheme 7:**
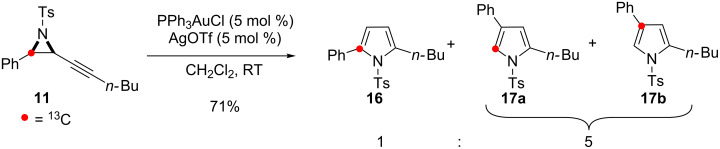
Cycloisomerisation of **11** in the skeletal rearrangement reaction.

**Scheme 8:**
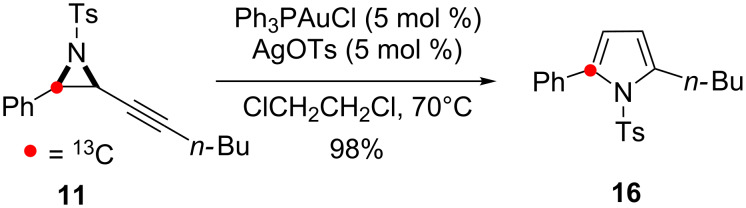
Cycloisomerisation of **11** to give 2,5-disubstituted pyrrole.

On subjecting **11** to the conditions for skeletal rearrangement (Scheme 7), three enhanced resonances were seen in the ^13^C NMR spectra of the pyrrole products (Supporting Information File 1). One of the enhanced resonances was associated with the minor 2,5-disubstituted isomer **16** showing ^13^C enrichment at the expected C-2 position (Scheme 2, Path I). This result was confirmed by carrying out the control cycloisomerisation of **11** under the conditions known to give solely 2,5-disubstituted products (Scheme 8) [19]. All other resonances were associated with the 2,4-disubstituted pyrrole **17** and the remaining enhancements were therefore assigned to the unexpected formation of two separate isotopomers of **17**. ^13^C-enrichment at C-5 (117.7 ppm) corresponds to the isotopomer **17a** anticipated from the proposed 1,2-aryl shift mechanism. Isotopomer **17b** was identified by enrichment at the quaternary C-4 position (127.0 ppm). HMBC experiments confirmed this assignment showing clear ^3^*J* coupling between the C-4 ^13^C-enriched quaternary centre and the protons of the phenyl ring.

**Scheme 9:**
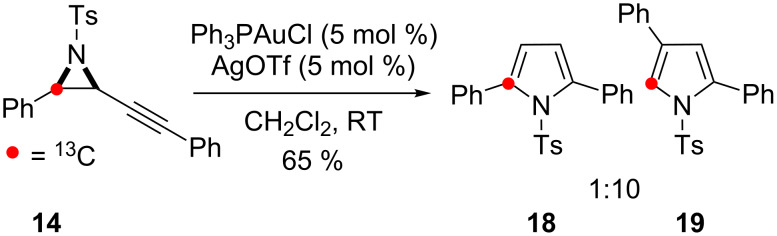
Cycloisomerisation of **14** in the skeletal rearrangement reaction.

Cycloisomerisation of the alkynyl aziridine **14**, which bears a phenyl substituent on the alkyne, resulted in a different outcome under the same reaction conditions: Only a single isotopomer of the 2,4-disubstituted pyrrole **19** was formed with ^13^C-enrichment at the C-5 position, alongside the 2,5-disubstituted isomer **18** (Scheme 9).

**Scheme 10:**
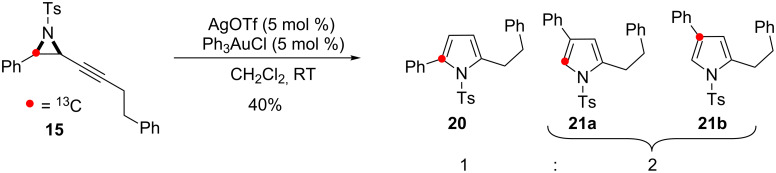
Cycloisomerisation of **15** in the skeletal rearrangement reaction.

The observed inconsistency between the reactions of **11** and **14** was investigated using alkyl substituted alkynyl aziridine **15**. Cycloisomerisation of **15** once again afforded a mixture of isotopomers of the 2,4-disubstituted pyrrole **21a**/**b** alongside 2,5-disubstituted pyrrole **20** (Scheme 10).

The formation of the isotopomers **17a, 19** and **21a** with ^13^C-enrichment at C-5 confirms C–C bond fission between the aryl group and the aziridine during the reaction. This outcome is consistent with an operative 1,2-aryl shift in the skeletal rearrangement of alkynyl aziridines to form 2,4-disubstituted pyrroles (Scheme 2, Path II).

However, the initial use of the alkyl substituted aziridine **11** rather than the aryl-substituted aziridine **14** was fortuitous. A second accessible pathway must exist to account for the formation of the 2,4-disubstituted pyrrole isotopomers **17b**, and **21b** by an alternate skeletal rearrangement.

**Scheme 11:**
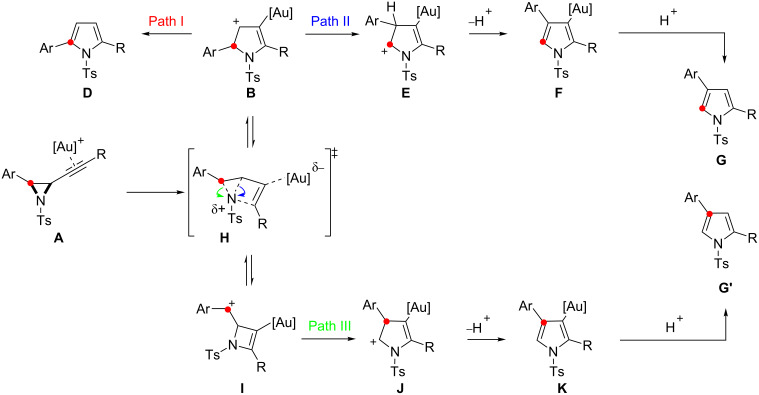
Revised mechanism for the formation of 2,4-isomers by skeletal rearrangement.

The mechanistic proposal for the skeletal rearrangement was therefore reappraised to explain these results (Scheme 11). Alongside the ring expansion of metal coordinated alkynyl aziridines to the 5-membered cyclic cation **B**, which leads to isotopomer **G** (Path II) and 2,5-disubstituted pyrroles **D** (Path I), a competing regioisomeric ring-expansion could afford 4-membered ring intermediate **I**. Evolution of **I** by a ring-expanding 1,2-shift of the metal stabilised vinyl unit onto the benzylic cation leads to a new 5-membered cyclic intermediate **J** [29]. Deprotonation and protodemetallation of **J** results in a 2,4-disubstituted isomer **G’** where the ^13^C-enriched carbon maintains its connectivity to the aryl unit, and C–C bond fission occurs between the aziridine and the alkyne fragment.

The observed effects of the alkyne substituent can be rationalised if the pathway is dependent on the relative stabilisation of the developing positive charge at either carbon of the aziridine by the alkynyl aziridine intermediate **H** (Scheme 11). The formation of either benzylic cation **I** (Path III) or the enamide stabilised cation **B** (Path II), under a cationic ring-expansion manifold, is apparently closely balanced: Products from both pathways are observed for alkyl substituted systems such as **11** and **15**. However, when the alkyne substituent is a phenyl group Path II is favoured over Path III due to the additional stabilisation of **B** by delocalisation through an extended π-system.

The initial report showed how the ratio of 2,5- to 2,4-disubstituted pyrroles varied with the electronic properties of the aromatic unit substituent directly attached to the aziridine [18]. While the trend was consistent with that expected for a 1,2-aryl shift, the more complex mechanism also accounts for the observed outcomes. An increasingly electron-rich aryl group will favour both formation of benzylic cation **I** and 1,2-aryl migration from **B**, leading to the same products from each route. Electron-deficient aromatics would have a destabilising effect on the formation of **I** and also be less prone to undergo 1,2-aryl shift from **B** thus favouring Path I and formation of the 2,5-disubstituted pyrroles.

Two further ^13^C labelled compounds were prepared to test this rationalisation by predictably affecting the relative stability of just one of the key intermediates: An electron-deficient aromatic substituent to the alkyne (R = *p*-C_6_H_4_CF_3_) should disfavour Path II by destabilising **B** over **I**, whereas an electron-rich aromatic substituent on the alkyne (R = *p*-C_6_H_4_OMe) should favour Path II by further stabilising **B**.

**Scheme 12:**
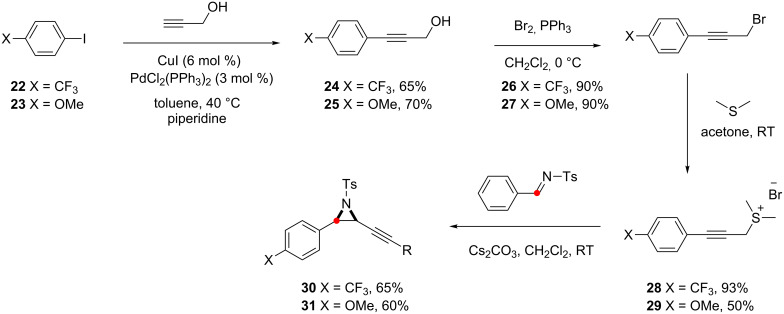
Synthesis of alkynyl aziridines **30** and **31**.

The substrates were prepared by Sonogashira reaction between propargyl alcohol and the appropriately substituted iodobenzene substrate **22**, **23** (Scheme 12). Functional group interconversion of the alcohols by bromination and substitution results in the sulfonium salts **28**, **29** which were reacted with ^13^C labelled imine **10** to yield the desired alkynyl aziridines **30** and **31**.

**Scheme 13:**
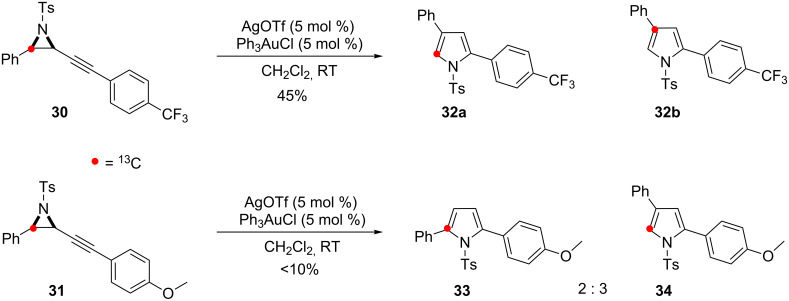
Electronic effects on the outcome of the skeletal rearrangement processes.

The anticipated outcomes of the catalysis were borne out under the standard conditions (Scheme 13). Two isotopomers of ^13^C-enriched 2,4-disubstituted pyrrole **32** were produced from the reaction of **30**. Qualitative analysis of the two isotopomers showed that a greater proportion of the 2,4-isomer formed from Path III was observed than with the other cyclisation precursors (Supporting Information File 1). Additionally, none of the 2,5-disubstituted pyrrole isomer, which would also result from **B**, was formed.

In keeping with the previous observation that strongly electron-rich aromatic groups were poor substituents for this cycloisomerisation [19], only a low yield of the pyrrole products was obtained on reaction of **31**. However, this was sufficient to determine that Path II predominated over Path III, with no significant isotopomer identified from intermediate **I**. Isomer **34** was identified alongside a significant quantity of the 2,5-disubstituted pyrrole **33**. While solvents and/or counter ions that stabilise positive charge favour the formation of the 2,5-disubstituted pyrroles, likewise, reducing the localised cationic charge in the 5-ring intermediate **B** by conjugation appears to have the same effect.

**Scheme 14:**
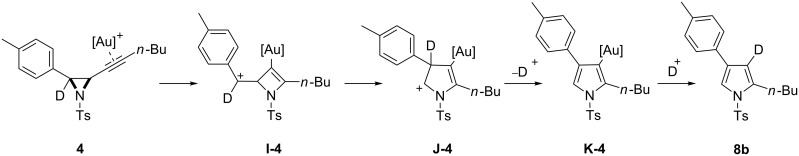
Mechanistic rationale for the deuterium labelling study using Ph_3_PAuCl/AgOTf.

Re-evaluation of the cycloisomerisation of the deuterium-labelled alkynyl aziridine **4** (Scheme 4) shows the results to be consistent with both the modified mechanistic scenario and the trends shown in the ^13^C-labelling study. Relative to all the other substrates tested, excepting perhaps **30**, substrate **4** would be the most likely to favour Path III over Path II (Scheme 11). Whilst the butyl substituent does not lend any additional stabilisation to intermediate **B**, the tolyl group stabilises the benzylic cation **I** better than a phenyl group. Significant deuterium incorporation at C-5 in the product would therefore not be expected. Following Path III, the deuterium labelled carbon ends-up at C-4 (Scheme 14, **I-4**→**J-4**). Aromatisation results in C–D bond cleavage with deuterodemetallation of **K-4** affording the observed major product **8b** (Scheme 5). The intermolecular deuteron transfer allows for H/D exchange in the reaction media and hence the partial incorporation of a proton at C-3.

## Conclusion

There are two operative skeletal rearrangement pathways for the formation of 2,4-disubstituted pyrroles by gold-catalysed cycloisomerisation of aryl-substituted *N*-tosyl alkynyl aziridines. The two competing pathways coincidentally give rise to the same isomer but with different absolute connectivities, as determined by the formation of two ^13^C isotopomeric products.

A modified mechanistic scheme accounts for all of the results of the labelling studies. On activation by a cationic gold source and in the absence of coordinating solvents and/or counter ions, regioisomeric ring expansion of the alkynyl aziridine can lead to two different cationic cyclic intermediates, featuring either a 4-membered or a 5-membered ring. The regioselectivity, and hence the isotopomeric reaction selectivity, is controlled by the relative ability of the substituents to stabilise the respective cations in favour of a particular pathway. The observed skeletal rearrangements are consistent with either a 1,2-aryl shift or a 1,2-(metal-stabilised)-vinyl shift in the 5- or 4-membered cyclic intermediates, respectively. These studies highlight some of the complexities associated with gold-catalysed cycloisomerisation reactions and the continued relevance of labelling studies in this field.

## Supporting Information

Supporting Information contains full experimental details for the preparation of the cyclisation precursors and their subsequent reactions. NMR spectra for the labelling studies are provided.

File 1 Full experimental details.
