# Supplementary material for: Isotopic labelling studies for a gold-catalysed skeletal rearrangement of alkynyl aziridines
Source: Beilstein J Org Chem. 2011 Jun 21;7:839–46. doi: 10.3762/bjoc.7.96 (PMC3135223; doi:10.3762/bjoc.7.96)
Supplement: File 1 — Full experimental details. [file Beilstein_J_Org_Chem-07-839-s001.pdf]

Supporting Information

for

Isotopic labelling studies for a gold-catalysed skeletal rearrangement of alkynyl aziridines

Paul W. Davies*, Nicolas Martin and Neil Spencer

Address: School of Chemistry, University of Birmingham, Edgbaston, Birmingham, B15 2TT, United Kingdom

Email: Paul Davies - p.w.davies@bham.ac.uk

* Corresponding author

Full experimental details

Contents

General Experimental S2

Procedures and Characterisation...S3

Preparation of alkynyl aziridines S3

Cycloisomerisation experiments using D-labelled alkynyl aziridine **4** S11

Cycloisomerisation experiments using ^{13}C-labelled alkynyl aziridine **11**........................S12

Cycloisomerisation experiment using ^{13}C-labelled alkynyl aziridine **14** S18

Cycloisomerisation experiment using ^{13}C-labelled alkynyl aziridine **15** S20

Cycloisomerisation experiments using ^{13}C-labelled alkynyl aziridine **30**........................S23

Cycloisomerisation experiments using ^{13}C-labelled alkynyl aziridine **31**........................S25

General Experimental

Asynt DrySin heating blocks on stirrer hotplates were employed for reactions with temperature control via an external probe. Infrared spectra were recorded neat on a Perkin–Elmer Spectrum 100 FT-IR spectrometer. Only selected absorbencies (ν_{max}) are reported, in cm^{-1}. High resolution mass spectra (HRMS) were recorded on a VG ProSpec or a VG-ZabSpec at 70 eV when utilising electron impact ionisation (EI). A Micromass LCT using a methanol mobile phase was used for HRMS utilising electrospray ionisation. In both cases (EI or ES), HRMS was obtained using a lock-mass to adjust the calibrated mass scale. MS data are reported as *m*/*z*. NMR: Spectra were recorded on Bruker AC300 (^{1}H = 300 MHz, ^{13}C = 75.5 MHz), Bruker AV300 (^{1}H = 300 MHz, ^{13}C = 75.5 MHz) or Bruker AV400 (^{1}H = 400 MHz, ^{13}C = 101 MHz), in the solvents indicated; Chemical shifts (δ) are given in ppm relative to TMS. Solvent signals were used as references and the chemical shifts converted to the TMS scale ($CDCl_3$: $\delta_C \equiv 77.0$ ppm; $CHCl_3$ in $CDCl_3$: $\delta_H \equiv 7.26$ ppm). Coupling constants (*J*) are reported in Hz. Multiplicity is denoted in ^{1}H NMR by: s (singlet), d (doublet), t (triplet), q (quadruplet) and m (multiplet). ^{13}C NMR spectra were recorded using the PENDANT pulse sequence from the Bruker standard pulse program library. Melting points were recorded in open glass capillaries on a Stuart Scientific apparatus and are uncorrected.

Reactions were followed by thin layer chromatography (TLC) using Macherey Nagel silica gel 60F254 analytical plates (plastic support) which were developed using standard visualizing agents: UV fluorescence (254 and 366 nm), and potassium permanganate/Δ. Purification by flash chromatography was performed on Fluorochem silica gel 60 (0.043–0.063 mm). All reactions in non-aqueous solvents were conducted in flame-dried glassware under an argon atmosphere and with magnetic stirring. Volumes of less than 0.2 mL were measured and dispensed with gas tight syringes. Evaporation and concentration under reduced pressure was performed at 10–700 mbar at 40 °C. All pure products of reactions were dried under high vacuum (<1 mbar).

All reagents were obtained from commercial sources and used without further purification.

The solvents used were purified by distillation over the drying agents indicated and were transferred under argon: Diethyl ether (sodium benzophenone ketyl), toluene (sodium), dichloromethane (CaH_2) and dichloroethane (CaH_2). Dess-Martin periodinane was prepared from 2-iodoxybenzoic acid (IBX) [1] following a known procedure [2].

Procedures and Characterisation

Preparation of alkynyl aziridines

Formation of aziridines from imine and sulfonium salt: General Procedure 1 (GP1):

The corresponding sulfonium salt (1.2 mmol) and Cs_2CO_3 (1.2 mmol) were added sequentially to a solution of the imine (1.0 mmol) in CH_2Cl_2 (10 mL). The reaction mixture was stirred at room temperature until completion and filtered through a pad of silica to remove the inorganic salts. The filtrate was then concentrated under reduced pressure and the residue purified by flash chromatography to afford the alkynyl aziridine.

Sulfonium salt preparation: General procedure 2 (GP2)

Dimethyl sulfide (15.0 mmol, 932 mg, 1.1 mL) was added to a solution of the bromide (5 mmol) in acetone (5 mL) and the reaction mixture stirred at room temperature for 3 days. A white solid was formed which was removed by filtration, washed with diethyl ether (4 × 10 mL) and dried to afford the corresponding pure sulfonium salt.

Sonogashira coupling of aryliodides with propargyl alcohol: General procedure 3 (GP3)

$Pd(PPh_3)_2Cl_2$ (0.75 mmol, 530 mg), CuI (1.5 mmol, 285 mg) and piperidine (47.8 mmol, 4.72 mL) were added to a solution of aryl iodide (25 mmol) in toluene (30 mL) at room temperature After stirring for 5 min at room temperature, propargyl alcohol (25.5 mmol, 1.48 mL) was added dropwise. The reaction mixture was then heated at 40 °C for 12 h. After cooling to room temperature, the reaction was filtered through a plug of silica and eluted with EtOAc. The filtrate was concentrated under reduced pressure and purification of the residue by flash chromatography gave the pure propargylic alcohol.

Preparation of propargylic bromides from propargylic alcohols: General procedure 4 (GP4)

Bromine (1.9 equiv) was added dropwise to a solution of PPh_3 (1.1 equiv) in CH_2Cl_2 at 0 °C. The reaction mixture was stirred for 20 min at 0 °C before a solution of the alcohol (1 equiv) in CH_2Cl_2 was added dropwise. The reaction mixture was warmed to room temperature and stirred for 1 h. Water (25 mL) was added to quench the reaction and the two phases were separated. The aqueous phase was washed with CH_2Cl_2 (3 × 20 mL) and the combined organic extracts were washed with brine (20 mL), dried (Na_2SO_4) and concentrated under

reduced pressure. Purification of the residue by flash chromatography gave the corresponding propargylic bromide.

4-Methylbenzaldehyde-α-*d*

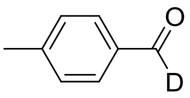

Ethyl 4-methylbenzoate **5** (2.5 mmol, 0.39 mL) was added to a suspension of $LiAlD_4$ (3.5 mmol, 147 mg) in Et_2O (5 mL) at 0 °C. The reaction mixture was stirred at room temperature for 1 h. After cooling to 0 °C, water (2.5 mL) was cautiously added to quench the reaction. A solution of HCl (10%, 2.5 mL) was added to solubilise the suspension and the two phases were separated. The aqueous phase was washed with Et_2O (3 × 10 mL). The combined organic abstracts were washed with brine (10 mL), dried (Na_2SO_4), and concentrated under reduced pressure to give 4-methylbenzyl alcohol-α,α-*d*.

A solution of the crude deuterated alcohol in CH_2Cl_2 (2 mL) was added to a solution of DMP (3 mmol, 1.26 g) in CH_2Cl_2 (5 mL). The reaction mixture was stirred at room temperature for 2 h and a solution of $Na_2S_2O_3$ (5 mL) added to quench the reaction. The two phases were separated and the aqueous phase was washed with CH_2Cl_2 (3 × 10 mL). The combined organic extracts were washed with brine (10 mL), dried (Na_2SO_4) and concentrated under reduced pressure. The residue was purified by distillation under reduced pressure (78 °C at 10 mmHg) to give the product as a colourless liquid (455 mg, 85%); δ_H (300 MHz; $CDCl_3$) 2.43 (3H, s, CH_3), 7.30 (2H, d, *J* 7.9, 2 × CH), 7.75 (2H, d, *J* 7.9, 2 × CH); δ_C (75 MHz; $CDCl_3$) 21.9 (CH_3), 129.7 (2C, 2 × CH), 129.9 (2C, 2 × CH), 134.2 (C_{quat}), 145.5 (C_{quat}).

***N*-(Deuteriophenylmethylene)-4-methylbenzenesulfonamide (6)**

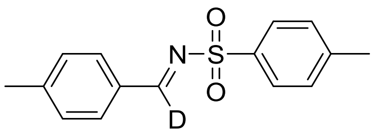

A mixture of 4-methylbenzaldehyde-α-*d* (2 mmol, 428 mg), *p*-toluenesulfonamide (1.9 mmol, 325 mg), amberlyst (150 mg) and 4 Å molecular sieve (150 mg) in toluene was stirred under reflux in a Dean–Stark apparatus. After 12 h, the reaction mixture was cooled to room temperature and filtered. The filtrate was concentrated under reduced pressure and the residue recrystallized from ethyl acetate/n-pentane to give imine **6** as a white solid (383 mg, 70%); 99–100 °C; ν_{max} (neat)/cm^{-1} 3356, 3260, 1582, 1552, 1508, 1494, 1445, 1409, 1387, 1318, 1303, 1288, 1155, 1089, 1033, 1018, 905, 858, 821, 809, 785, 753, 705; δ_H (300 MHz; $CDCl_3$) 2.43 (6H, s, 2 × CH_3), 7.28 (2H, d, *J* 7.9, 2 × CH), 7.34 (2H, d, *J* 7.9, 2 × CH), 7.81 (2H, d, *J* 8.2, 2 × CH), 7.88 (2H, d, *J* 8.2, 2 × CH); δ_C (75 MHz; $CDCl_3$) 21.6 (CH_3), 22.0 (CH_3), 128.0 (2C, 2 × CH), 129.8 (2C, 2 × CH), 129.9 (2C, 2 × CH), 131.4 (2C, 2 × CH),

135.5 (C_{quat}), 144.4 (2C, 2 × C_{quat}), 146.4 (2C, 2 × C_{quat}); HRMS *m*/*z* (TOF ES+) 297.0791. $C_{15}H_{14}DNO_2NaS$ requires 297.0784.

2-Deuterio-3-hex-1-ynyl-1-(toluene-4-sulfonyl)-2-*p*-tolylaziridine (4)

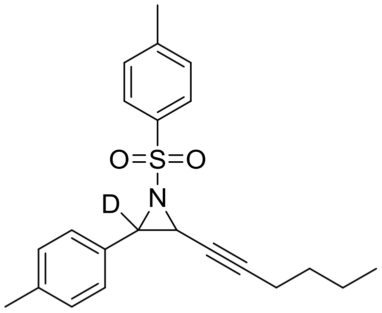

Following GP1 from imine **6** and sulfonium salt **7,** reaction time 3 h. Purification by flash chromatography [hexane:ethyl acetate (25:1)] gave aziridine **4** as a beige solid (143 mg, 77%, 14:1 *cis*:*trans*); ν_{max} (neat)/cm^{-1} 2961, 2926, 2874, 2248, 1921, 1598, 1518, 1458, 1410, 1363, 1323, 1301, 1181, 1161, 1133, 1090, 1019, 914, 894, 838, 805, 757, 704; δ_H (300 MHz; $CDCl_3$) 0.76 (3H, t, *J* 7.2, CH_3), 1.05–1.18 (2H, m, CH_2), 1.22–1.31 (2H, m, CH_2), 2.02 (2H, td, *J* 6.9 and 1.8, CH_2), 2.32 (3H, s, CH_3), 2.43 (3H, s, CH_3), 3.60 (1H, t, *J* 1.8, CH), 7.09 (2H, d, *J* 8.1, 2 × CH), 7.21 (2H, d, *J* 8.1, 2 × CH), 7.33 (2H, d, *J* 8.4, 2 × CH), 7.87 (2H, d, *J* 8.4, 2 × CH); δ_C (75 MHz; $CDCl_3$) 13.4 (CH_3), 18.3 (CH_2), 21.2 (CH_3), 21.5 (CH_2), 21.6 (CH_3), 30.0 (CH_2), 36.1 (CH), 72.3 (C_{quat}), 86.6 (C_{quat}), 127.6 (2C, 2 × CH), 127.9 (2C, 2 × CH), 128.6 (2C, 2 × CH), 129.1 (C_{quat}), 129.7 (2C, 2 × CH), 134.8 (C_{quat}), 138.0 (C_{quat}), 144.7 (C_{quat}); HRMS *m*/*z* (TOF ES+) 391.1563. $C_{22}H_{24}DNO_2NaS$ requires 391.1566.

^{13}C-enriched benzaldehyde

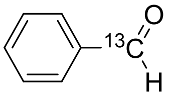

A solution of ^{13}C-enriched benzoic acid (5 mmol, 610 mg, ^{13}C:^{12}C 1:5) in Et_2O (5 mL), was added dropwise to a suspension of $LiAlH_4$ (12 mmol, 504 mg) in Et_2O (25 mL) at 0 °C. After 20 min stirring the reaction mixture was heated at 50 °C for 2 h. After cooling to 0 °C, water (15 mL) was cautiously added to quench the reaction. A solution of HCl (10%, 5 mL) was added to solubilise the suspension and the two phases were separated. The aqueous phase was washed with Et_2O (3 × 15 mL). The combined organic extracts were washed with brine (15 mL), dried (Na_2SO_4), and concentrated under reduced pressure to give ^{13}C-enriched benzyl alcohol.

A solution of the crude ^{13}C-enriched benzyl alcohol in CH_2Cl_2 (5 mL) was added to a solution of DMP (7.5 mmol, 3.16 g) in CH_2Cl_2 (30 mL). The reaction mixture was stirred at room temperature for 4 h and a solution of $Na_2S_2O_3$ (15 mL) added to quench the reaction. The two phases were separated and the aqueous phase was washed with CH_2Cl_2 (3 × 15 mL). The combined organic extracts were washed with brine (15 mL), dried (Na_2SO_4) and concentrated under reduced pressure. The residue was purified by distillation under reduced

pressure (75 °C at 10 mmHg) to give ^{13}C-enriched benzaldehyde as a colourless liquid (425 mg, 80%); δ_H (300 MHz; $CDCl_3$) 7.50–7.73 (3H, m, 3 × CH), 7.75–7.92 (2H, m, 2 × CH), 10.07 (1H, s, CH); δ_C (75 MHz; $CDCl_3$) 129.2 (2C, 2 × CH), 130.0 (2C, 2 × CH), 134.7 (CH), 136.7 (C_{quat}), 192.6 (^{13}C-enriched signal, C_{quat}).

^{13}C-enriched *N*-benzylidene-4-methylbenzenesulfonamide (10)

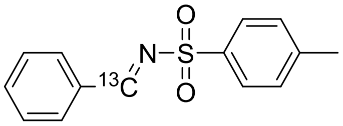

A 1:5 mixture of benzaldehyde-α-^{13}C and benzaldehyde (5.5 mmol, 589 mg), *p*-toluenesulfonamide (5.0 mmol, 856 mg), amberlyst 15 (380 mg) and 4Å molecular sieve (380 mg) in toluene (30 mL) was stirred at 130 °C in a Dean-Stark apparatus. After 12 h, the reaction mixture was cooled to room temperature and filtered. The filtrate was concentrated under reduced pressure and the residue was recrystallized from ethyl acetate/*n*-pentane to give ^{13}C-enriched imine **10** as a white solid (907 mg, 70%); mp 102–103 °C; ν_{max} (neat)/cm^{-1} 2922, 2853, 2179, 1598, 1449, 1413, 1364, 1326, 1291, 1245, 1158, 1135, 1090, 1061, 994, 959, 859, 838, 824, 815, 749, 701; δ_H (300 MHz; $CDCl_3$) 2.41 (3H, s, CH_3), 7.35 (2H, d, *J* 8.0, 2 × CH), 7.49 (2H, d, *J* 8.0, 2 × CH), 7.59–7.64 (1H, m, CH), 7.88–7.94 (4H, m, 4 × CH), 9.03 (1H, s, CH); δ_C (75 MHz; $CDCl_3$) 21.6 (CH_3), 128.0 (2C, 2 × CH), 129.1 (2C, 2 × CH), 129.8 (2C, 2 × CH), 131.3 (2C, 2 × CH), 132.4 (C_{quat}), 134.9 (CH), 135.1 (C_{quat}), 144.6 (C_{quat}), 170.1 (^{13}C-enriched signal, CH); HRMS *m*/*z* (TOF ES+) 283.0592. $C_{13}{}^{13}CH_{13}NO_2NaS$ requires 283.0598.

^{13}C-enriched 2-(hex-1-ynyl)-3-phenyl-1-(toluene-4-sulfonyl)aziridine (11)

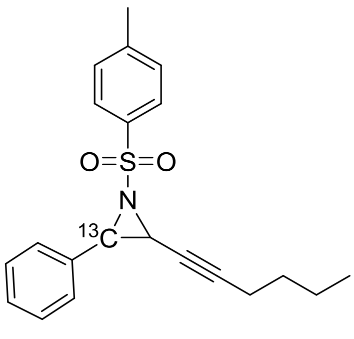

Following GP1 from ^{13}C-enriched imine **10** and sulfonium salt **7** [3], reaction time 3 h. Purification by flash chromatography [hexane:ethyl acetate (12:1)] gave ^{13}C-enriched aziridine **11** as a white solid (212 mg, 60%, 8:1 *cis*:*trans*); ν_{max} (neat)/cm^{-1} 2960, 2934, 2252, 1601, 1497, 1455, 1381, 1319, 1305, 1292, 1230, 1187, 1158, 1088, 1038, 1025, 871, 811, 784, 754, 738, 717,695, 672; δ_H (300 MHz; $CDCl_3$) 0.75 (3H, t, *J* 7.2, CH_3), 1.01–1.32 (4H, m, 2 × CH_2), 1.98 (2H, td, *J* 6.8 and 1.7, CH_2), 2.42 (3H, s, CH_3), 3.63 (1H, dt, *J* 6.9 and 1.7, CH), 3.94 (1H, d, *J* 6.9, CH), 7.21–7.39 (7H, m, 7 × CH), 7.88 (2H, d, *J* 8.3, 2 × CH); δ_C (75 MHz; $CDCl_3$) 13.4 (CH_3), 18.2 (CH_2), 21.4 (CH_2), 21.6 (CH_3), 30.0 (CH_2), 36.2 (CH), 46.1 (^{13}CH, enriched signal, CH), 72.1 (C_{quat}), 86.7 (C_{quat}), 127.7 (2C, 2 × CH) 127.9 (4C, 4 × CH), 128.2

(CH), 129.8 (2C, 2 × CH), 132.2 (C_{quat}), 134.7 (C_{quat}), 144.6 (C_{quat}); HRMS *m*/*z* (TOF ES+) 377.1376. $C_{20}{}^{13}CH_{23}NO_2NaS$ requires 377.1381.

^{13}C-enriched 2-phenyl-3-(phenylethynyl)-1-(toluene-4-sulfonyl)aziridine (14)

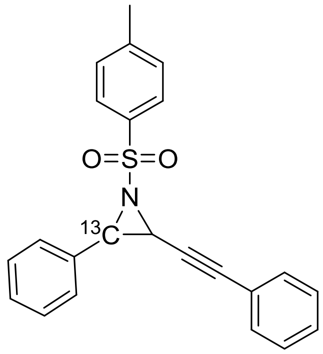

Following GP1 from ^{13}C-enriched imine **10** and sulfonium salt **12** [3], reaction time 1.5 h. Purification by flash chromatography [hexane:ethyl acetate (25:1)] gave ^{13}C-enriched aziridine **14** (298 mg, 80%, 12:1 *cis*:*trans*); ν_{max} (neat)/cm^{-1} 3032, 2950, 2926, 2240, 1597, 1490, 1457, 1441, 1319, 1157, 1087, 1071, 873, 854, 784, 757, 708; δ_H (300 MHz; $CDCl_3$) 2.44 (3H, s, CH_3), 3.87 (1H, d, *J* 6.9, CH), 4.09 (1H, d, *J* 6.9, CH), 7.12–7.28 (4H, m, 4 × CH), 7.29–7.45 (8H, m, 8 × CH), 7.92 (2H, d, *J* 8.3, 2 × CH); δ_C (75 MHz; $CDCl_3$) 21.7 (CH_3), 36.3 (CH), 46.5 (^{13}C-enriched signal, CH), 81.6 (C_{quat}), 85.1 (C_{quat}), 121.8 (C_{quat}), 127.8 (2C, 2 × CH), 128.0 (4C, 4 × CH), 128.1 (2C, 2 × CH), 128.5 (CH), 128.8 (CH), 129.9 (2C, 2 × CH), 131.8 (2C, 2 × CH), 132.1 (C_{quat}), 134.6 (C_{quat}), 144.9 (C_{quat}); HRMS *m*/*z* (TOF ES+) 397.1074. $C_{22}{}^{13}CH_{18}NO_2NaS$ requires 397.1068.

^{13}C-enriched 2-phenyl-3-(4-phenylbut-1-yn-1-yl)-1-(toluene-4-sulfonyl)aziridine (15)

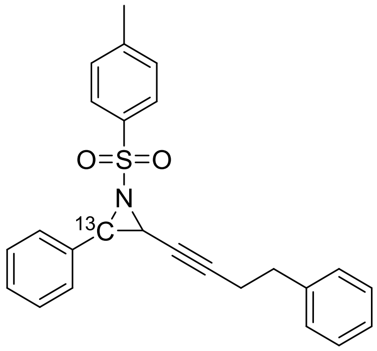

Following GP1 from ^{13}C-enriched imine **10** and sulfonium salt **13** [3], reaction time 4 h. Purification by flash chromatography [hexane:ethyl acetate (20:1)] gave ^{13}C-enriched aziridine **15** (269 mg, 67%, 14:1 *cis*:*trans*). ν_{max} (neat)/cm^{-1} 3029, 2925, 2248, 1597, 1495, 1454, 1384, 1327, 1291, 1235, 1158, 1090, 1021, 875, 814, 783, 742, 695; δ_H (300 MHz; $CDCl_3$) 2.27–2.33 (2H, m, CH_2), 2.44 (3H, s, CH_3), 2.50–2.65 (2H, m, CH_2), 3.62 (1H, dt, *J* 6.9 and 1.8, CH), 3.94 (1H, d, *J* 6.9, CH), 6.95–7.01 (2H, m, 2 × CH), 7.14–7.24 (3H, m, 3 × CH), 7.29 (5H, s,5 × CH), 7.34 (2H, d, *J* 8.3, 2 × CH), 7.88 (2H, d, *J* 8.3, 2 × CH); δ_C (75 MHz; $CDCl_3$) 20.8 (CH_2), 21.7 (CH_3), 34.1 (CH_2), 36.1 (CH), 46.1 (^{13}C-enriched signal, CH), 73.0 (C_{quat}), 85.8 (C_{quat}), 126.2 (2C, 2 × CH), 127.8 (2C, 2 × CH), 127.9 (2C, 2 × CH), 128.0 (2C, 2 × CH), 128.3 (5C, 5 × CH), 129.8 (2C, 2 × CH), 132.2 (C_{quat}), 134.7 (C_{quat}), 140.2 (C_{quat}), 144.8 (C_{quat}); HRMS *m*/*z* (TOF ES+) 425.1373. $C_{24}{}^{13}CH_{23}NO_2NaS$ requires 425.1381.

3-(4-Trifluoromethylphenyl)prop-2-yn-1-ol (24) [4]

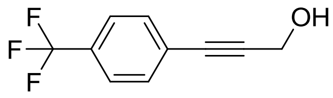

Following GP3 from 1-iodo-4-(trifluoromethyl)benzene (6.80 g, 3.67 mL). Purification by flash chromatography [hexane:ethyl acetate (4:1)] gave alcohol **24** as a brown solid (2.75 g, 90%); ν_{max} (neat)/cm^{-1} 3350, 3080, 2890, 2275, 1622, 1532, 1405, 1328, 1186, 1129, 953, 786, 732, 689; δ_H (300 MHz; $CDCl_3$) 1.91 (1H, t, *J* 5.5, OH), 4.54 (2H, d, *J* 5.5, CH_2), 7.50 (2H, d, *J* 8.5, 2 × CH), 7.54 (2H, d, *J* 8.5, 2 × CH); δ_C (75 MHz; $CDCl_3$) 51.5 (CH_2), 84.3 (C_{quat}), 89.6 (C_{quat}), 122.5 (q, *J* 272.2, C_{quat}), 125.2 (2C, q, *J* 3.5, 2 × CH), 126.4 (C_{quat}), 130.2 (q, *J* 32.9, C_{quat}), 131.9 (2C, 2 × CH).

3-(4-Methoxyphenyl)prop-2-yn-1-ol (25) [5]

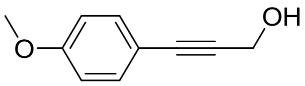

Following GP3 using 4-iodoanisole (5.85 g). Purification by flash chromatography [hexane:ethylacetate (2:1)] gave alcohol **25** as a brown solid (2.64 g, 65%);δ_H (300 MHz; $CDCl_3$) 1.83 (1H, s, OH), 3.81 (3H, s, CH_3), 4.49 (2H, s, CH_2), 6.85 (2H, d, *J* 6.5, 2 × CH), 7.40 (2H, d, *J* 6.5, 2 × CH); δ_C (75 MHz; $CDCl_3$) 51.7 (CH_2), 55.3 (CH_3), 85.7 (C_{quat}), 85.9 (C_{quat}), 114.0 (2C, 2 × CH), 114.6 (C_{quat}), 133.2 (2C, 2 × CH), 159.8 (C_{quat}); HRMS *m*/*z* (TOF EI+) 162.0683. $C_{10}H_{10}O_2$ requires 162.0681.

1-(3-Bromoprop-1-ynyl)-4-(trifluoromethyl)benzene (26) [6]

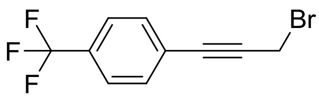

Following GP4 using PPh_3 (11 mmol, 2.88 g), Br_2 (10.9 mmol, 0.55 mL) and alcohol **24** (2.00 g) in CH_2Cl_2 (30 mL). Purification by flash chromatography (*n*-pentane) gave bromine **26** as a yellow oil (2.36 g, 90%); ν_{max} (neat)/cm^{-1} 3012, 2232, 2199, 1930, 1725, 1669, 1516, 1407, 1423, 1329, 1129, 1073, 1052, 1022, 850, 769; δ_H (300 MHz; $CDCl_3$) 4.16 (2H, s, CH_2), 7.54 (2H, d, *J* 8.4, 2 × CH), 7.57 (2H, d, *J* 8.4, 2 × CH); δ_C (75 MHz; $CDCl_3$) 14.4 (CH_2), 85.1 (C_{quat}), 86.6 (C_{quat}), 123.9 (q, *J* 272.2, C_{quat}), 125.3 (2C, q, *J* 3.8, 2 × CH), 125.9 (C_{quat}), 130.6 (q, *J* 32.8, C_{quat}), 132.1 (2C, 2× CH); HRMS *m*/*z* (TOF EI+) 261.9590. $C_{10}H_6{}^{79}BrF_3$ requires 261.9605.

1-(3-Bromoprop-1-ynyl)-4-methoxybenzene (27) [7]

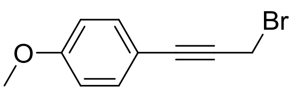

Following GP4 from PPh_3 (11 mmol, 2.88 g), Br_2 (10.9 mmol, 0.55 mL) and alcohol **25** (1.62 g) in CH_2Cl_2 (30 mL). Purification by flash chromatography (hexane) gave bromine **27** as a colourless oil (2.02 g, 90%); ν_{max} (neat)/cm^{-1} 2228, 1609, 1602, 1518, 1471, 1102, 1001, 960, 820; δ_H (300 MHz; $CDCl_3$) 3.79

(3H, s, CH_3), 4.17 (2H, s, CH_2), 6.84 (2H, d, *J* 9.0, 2 × CH), 7.39 (2H, d, *J* 9.0, 2 × CH); δ_C (75 MHz; $CDCl_3$) 16.0 (CH_2), 55.3 (CH_3), 83.0 (C_{quat}), 86.9 (C_{quat}), 114.0 (2C, 2 × CH), 114.1 (C_{quat}), 133.5 (2C, 2 × CH), 160.0 (C_{quat}).

Dimethyl(3-(4-(trifluoromethyl)phenyl)prop-2-yn-1-yl)sulfonium bromide (28)

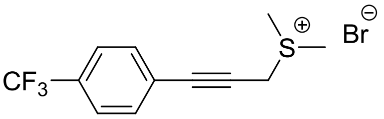

Following GP2 from bromide **26** (1.315 g) gave sulfonium salt **28** (1.625 g, 50%); mp 139–140 °C; ν_{max} (neat)/cm^{-1} 3006, 2924, 2891, 2245, 1618,1407, 1319, 1231, 1162, 1126, 1107, 1067, 1045, 1017, 1001, 982, 840, 712; δ_H (300 MHz; $CDCl_3$) 3.36 (6H, s, 2 × CH_3), 5.43 (2H, s, CH_2), 7.60–7.70 (4H, m, 4 × CH); δ_C (75 MHz; $CDCl_3$) 24.9 (2C, 2 × CH_3), 33.7 (CH_2), 76.9 (C_{quat}), 89.5 (C_{quat}), 123.5 (q, *J* 272.7, C_{quat}), 124.3 (C_{quat}), 125.7 (2C, q, *J* 3.3, 2 × CH), 131.6 (q, *J* 33.6, C_{quat}), 132.5 (2C, 2 × CH); HRMS *m*/*z* (TOF ES+) 245.0607. $C_{12}H_{12}F_3S$ requires 245.0612.

3-(4-Methoxyphenyl)prop-2-yn-1-yl)dimethylsulfonium bromide (29)

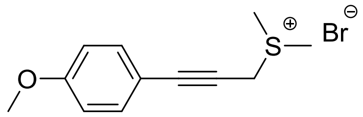

Following GP2 from bromide **27** (1.125 g) gave sulfonium salt **29** (1.335 g, 93%); mp 124–125 °C; ν_{max} (neat)/cm^{-1} 2969, 2907, 2864, 2216, 1605, 1565, 1509, 1459, 1421, 1325, 1296, 1276, 1246, 1180, 1169, 1105, 1046, 1021, 1009, 828, 800, 703; δ_H (300 MHz; $CDCl_3$) 3.31 (6H, s, 2 × CH_3), 3.81 (3H, s, CH_3), 5.31 (2H, s, CH_2), 6.85 (2H, d, *J* 8.8, 2 × CH), 7.40 (2H, d, *J* 8.8, 2 × CH); δ_C (75 MHz; $CDCl_3$) 24.6 (2C, 2 × CH_3), 34.2 (CH_2), 55.3 (CH_3), 73.0 (C_{quat}), 91.2 (C_{quat}), 112.5 (C_{quat}), 114.2 (2C, 2 × CH), 133.7 (2C, 2 × CH), 160.6 (C_{quat}); HRMS *m*/*z* (TOF ES+) 207.0844. $C_{12}H_{15}OS$ requires 207.0840.

^{13}C-enriched 2-phenyl-1-(toluene-4-sulfonyl)-3-((4-trifluoromethyl)phenyl)ethynyl)-aziridine (30)

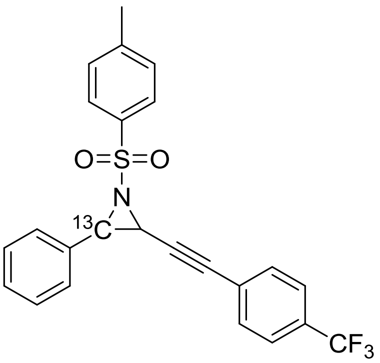

Following GP1 from ^{13}C-enriched imine **10** and sulfonium salt **28**, reaction time 30 min. Purification by flash chromatography [hexane:ethyl acetate (25:1)] gave ^{13}C-enriched aziridine **30** (287 mg, 65%, 15:1 *cis*:*trans*); ν_{max} (neat)/cm^{-1} 3065, 3012, 1617, 1596, 1496, 1456, 1405, 1378, 1320, 1157, 1127, 1106, 1088, 1059, 1016, 973, 870, 838, 812, 785, 737, 696; δ_H (300 MHz; $CDCl_3$) 2.44 (3H, s, CH_3), 3.90 (1H, d, *J* 6.8, CH), 4.13 (1H, d, *J* 6.8, CH), 7.27 (2H, d,

J 8.1, 2 × CH), 7.31–7.42 (7H, m, 7 × CH), 7.48 (2H, d, *J* 8.1, 2 × CH), 7.94 (2H, d, *J* 8.3, 2 × CH); δ_C (75 MHz; $CDCl_3$) 21.6 (CH_3), 35.8 (CH), 46.5 (^{13}C-enriched signal, CH), 83.5 (C_{quat}), 84.3 (C_{quat}), 123.6 (q, *J* 272.4, C_{quat}), 125.1 (2C, q, *J* 3.1, 2 × CH), 125.5 (C_{quat}), 127.7 (2C, 2 × CH), 128.0 (2C, 2 × CH), 128.1 (2C, 2 × CH), 128.6 (CH), 129.9 (2C, 2 × CH), 130.6 (q, *J* 32.7, C_{quat}), 131.9 (C_{quat}), 132.1 (2C, 2 × CH), 134.4 (C_{quat}), 145.1 (C_{quat}); HRMS *m*/*z* (TOF ES+) 465.0934. $C_{23}{}^{13}CH_{18}NO_2F_3NaS$ requires 465.0942.

^{13}C-enriched 2-((4-methoxyphenyl)ethynyl)-3-phenyl-1-(toluene-4-sulfonyl)aziridine (31)

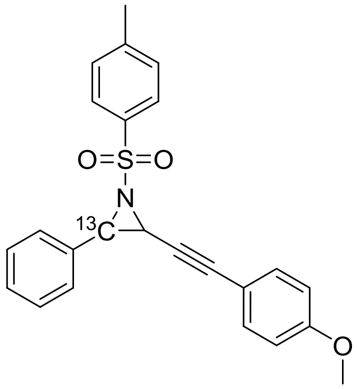

Following GP1 from ^{13}C-enriched imine **10** and sulfonium salt **29**, reaction time 45 min. Purification by flash chromatography [hexane:ethyl acetate (20:1)] gave ^{13}C-enriched aziridine **31** (242 mg, 60 %, 16:1 *cis*:*trans*); ν_{max} (neat)/cm^{-1} 3036, 2933, 2838, 2228, 1603, 1509, 1455, 1327, 1291, 1247, 1156, 1089, 1027, 976, 872, 831, 812, 786, 733, 697; δ_H (300 MHz; $CDCl_3$) 2.43 (3H, s, CH_3), 3.76 (3H, s, CH_3), 3.86 (1H, d, *J* 6.9, CH), 4.07 (1H, d, *J* 6.9, CH), 6.73 (2H, d, *J* 8.9, 2 × CH), 7.11 (2H, d, *J* 8.9, 2 × CH), 7.30–7.41 (7H, m, 7 × CH), 7.91 (2H, d, *J* 8.3, 2 × CH); δ_C (75 MHz; $CDCl_3$) 21.7 (CH_3), 36.5 (CH), 46.5 (^{13}C-enriched signal, CH), 55.2 (CH_3), 80.2 (C_{quat}), 85.3 (C_{quat}), 113.8 (2C, 2 × CH), 127.8 (2C, 2 × CH), 128.0 (5C, 5 × CH, C_{quat}), 128.4 (CH), 129.8 (2C, 2 × CH), 132.2 (C_{quat}), 133.4 (2C, 2 × CH), 134.7 (C_{quat}), 144.9 (C_{quat}), 159.9 (C_{quat}); HRMS *m*/*z* (TOF ES+) 426.1089. $C_{23}{}^{13}CH_{21}NO_3NaS$ requires 426.1095.

Cycloisomerisation experiments using D-labelled alkynyl aziridine 4

Gold-catalysed cycloisomerisations of alkynyl aziridines using $Ph_3PAuCl/AgOTf$: general procedure 5 (GP5):

The catalyst system was prepared by the addition of anhydrous CH_2Cl_2 (0.5 mL) to Ph_3PAuCl (0.01 mmol, 5.0 mg) and AgOTf (0.01 mmol, 2.5 mg) in a flame-dried Schlenk flask under an argon atmosphere. After stirring for 10 min at room temperature, a white precipitate of AgCl was observed and a solution of the corresponding alkynyl aziridine (0.2 mmol) in anhydrous CH_2Cl_2 (0.5 mL) was added. The reaction mixture was stirred at room temperature until complete consumption of aziridine before being filtered through a pad of silica. The filtrate was then concentrated under reduced pressure. The residue was purified by flash chromatography as indicated.

2-Butyl-3-deuterio-1-(toluene-4-sulfonyl)-4-*p*-tolyl-1*H*-pyrrole (8b)

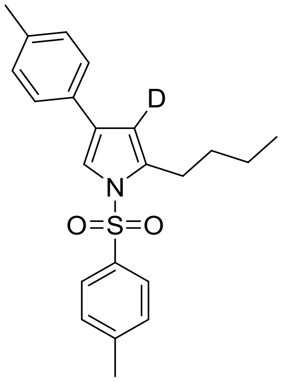

Following GP5 from aziridine **4** (74 mg), reaction time 1 h. Purification by flash chromatography [hexane:ethyl acetate (25:1)] gave a mixture of 2,4-disubstituted pyrrole **8b**, 2,4-disubstituted pyrrole **8c** and 2,5-disubstituted pyrrole **9** (13 mg, 18%, 10.5:3.1:1 **8b**:**8a**:**9**); δ_H (300 MHz; $CDCl_3$) 0.90 (3H, t, *J* 7.3, CH_3), 1.30–1.43 (2H, m, CH_2), 1.53–1.64 (2H, m, CH_2), 2.35 (3H, s, CH_3), 2.40 (3H, s, CH_3), 2.68 (2H, t, *J* 7.4, CH_2), 7.16 (2H, d, *J* 8.2, 2 × CH), 7.28 (2H, d, *J* 8.4, 2 × CH), 7.40 (2H, d, *J* 8.2, 2 × CH), 7.54 (1H, s, CH), 7.68 (2H, d, *J* 8.4, 2 × CH); δ_C (75 MHz; $CDCl_3$) 13.9 (CH_3), 21.1 (CH_3), 21.6 (CH_3), 22.4 (CH_2), 26.9 (CH_2), 30.7 (CH_2), 117.3 (CH), 125.3 (2C, 2 × CH), 126.7 (2C, 2 × CH),129.4 (2C, 2 × CH), 129.9 (2C, 2 × CH), 130.8 (C_{quat}), 136.4 (C_{quat}), 136.5 (C_{quat}), 136.6 (C_{quat}), 136.9 (C_{quat}), 144.7 (C_{quat}); HRMS *m*/*z* (TOF ES+) 391.1559. $C_{22}H_{24}DNO_2NaS$ requires 391.1566.

Cycloisomerisation experiments using ^{13}C-labelled alkynyl aziridine 11

Gold-catalysed cycloisomerisations of alkynyl aziridines using $Ph_3PAuCl/AgOTs$: general procedure 6 (GP6):

The catalyst system was prepared by the addition of anhydrous $ClCH_2CH_2Cl$ (0.5 mL) to Ph_3PAuCl (0.01 mmol, 5.0 mg) and AgOTs (0.01 mmol, 2.8 mg) in a flame-dried Schlenk flask under an argon atmosphere. After stirring for 10 min at room temperature, a white precipitate of AgCl was observed and a solution of the corresponding alkynyl aziridine (0.2 mmol) in anhydrous $ClCH_2CH_2Cl$ (0.5 mL) was added. The reaction mixture was stirred at the indicated temperature until aziridine was completely consumed, before being filtered through a pad of silica. The filtrate was then concentrated under reduced pressure. When required the residue was purified by flash chromatography as indicated.

^{13}C-enriched 2-butyl-5-phenyl-1-(toluene-4-sulfonyl)-1*H*-pyrrole (16)

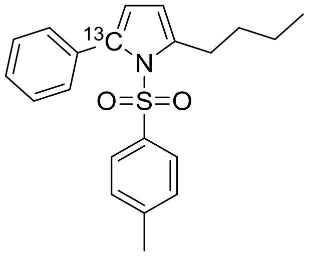

Following GP6 from ^{13}C enriched aziridine **11** (71 mg) at 70 °C for 4 h gave pyrrole **16** (69 mg, 98%); ν_{max} (neat)/cm^{-1} 3060, 2956, 2928, 2861, 1737, 1596, 1527, 1482, 1444, 1366, 1169, 1116, 1092, 911, 809, 759; δ_H (300 MHz; $CDCl_3$) 0.97 (3H, t, *J* 7.3, CH_3), 1.38–1.50 (2H, m, CH_2), 1.66–1.76 (2H, m, CH_2), 2.36 (3H, s, CH_3), 2.92 (2H, t, *J* 7.7, CH_2), 6.04 (1H, d, *J* 3.3, CH), 6.08 (1H, d, *J* 3.3, CH),7.14 (2H, d, *J* 8.4, 2 × CH), 7.28 (2H, d, *J* 8.4, 2 × CH), 7.32 (5H, s, 5 × CH); δ_C (75 MHz; $CDCl_3$) 14.0 (CH_3), 21.6 (CH_3), 22.5 (CH_2), 29.3 (CH_2), 31.6 (CH_2), 112.6 (CH), 115.6 (CH), 126.4 (2C, 2 × CH), 127.2 (2C, 2 × CH), 127.7 (CH), 129.3 (2C, 2 × CH), 130.4 (2C, 2 × CH), 133.3 (C_{quat}), 136.4 (C_{quat}), 138.0 (^{13}C-enriched signal, C_{quat}), 139.9 (C_{quat}), 144.2 (C_{quat}); HRMS *m*/*z* (TOF ES+) 377.1373. $C_{20}{}^{13}CH_{23}NO_2NaS$ requires 377.1381.

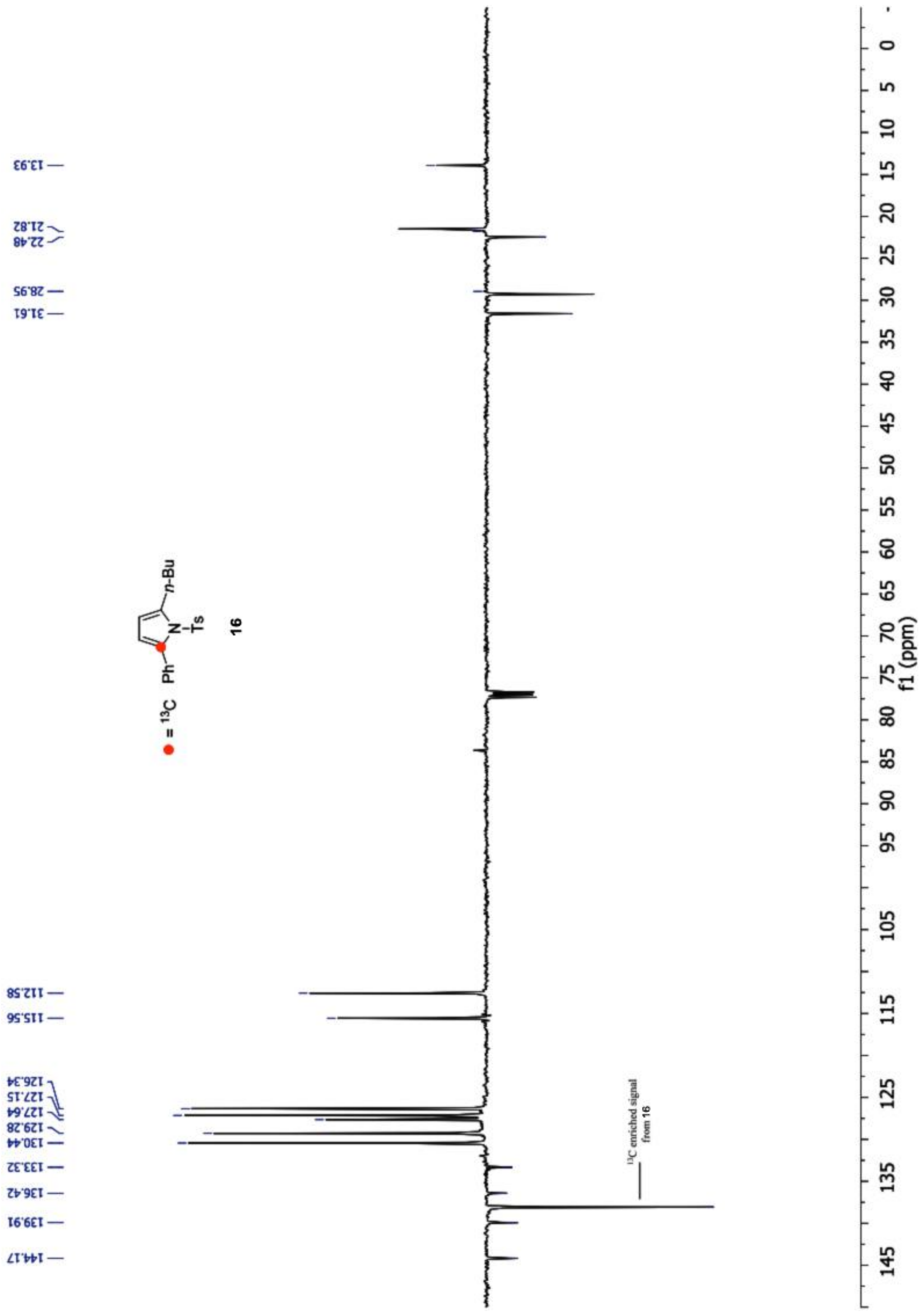
13.93
21.82
22.48
28.95
31.61
112.58
115.56
126.34
127.15
127.64
129.28
130.44
133.32
136.42
139.91
144.17
Ph
N
Ts
n-Bu
16
= 13C
13C enriched signal from 16
f1 (ppm)

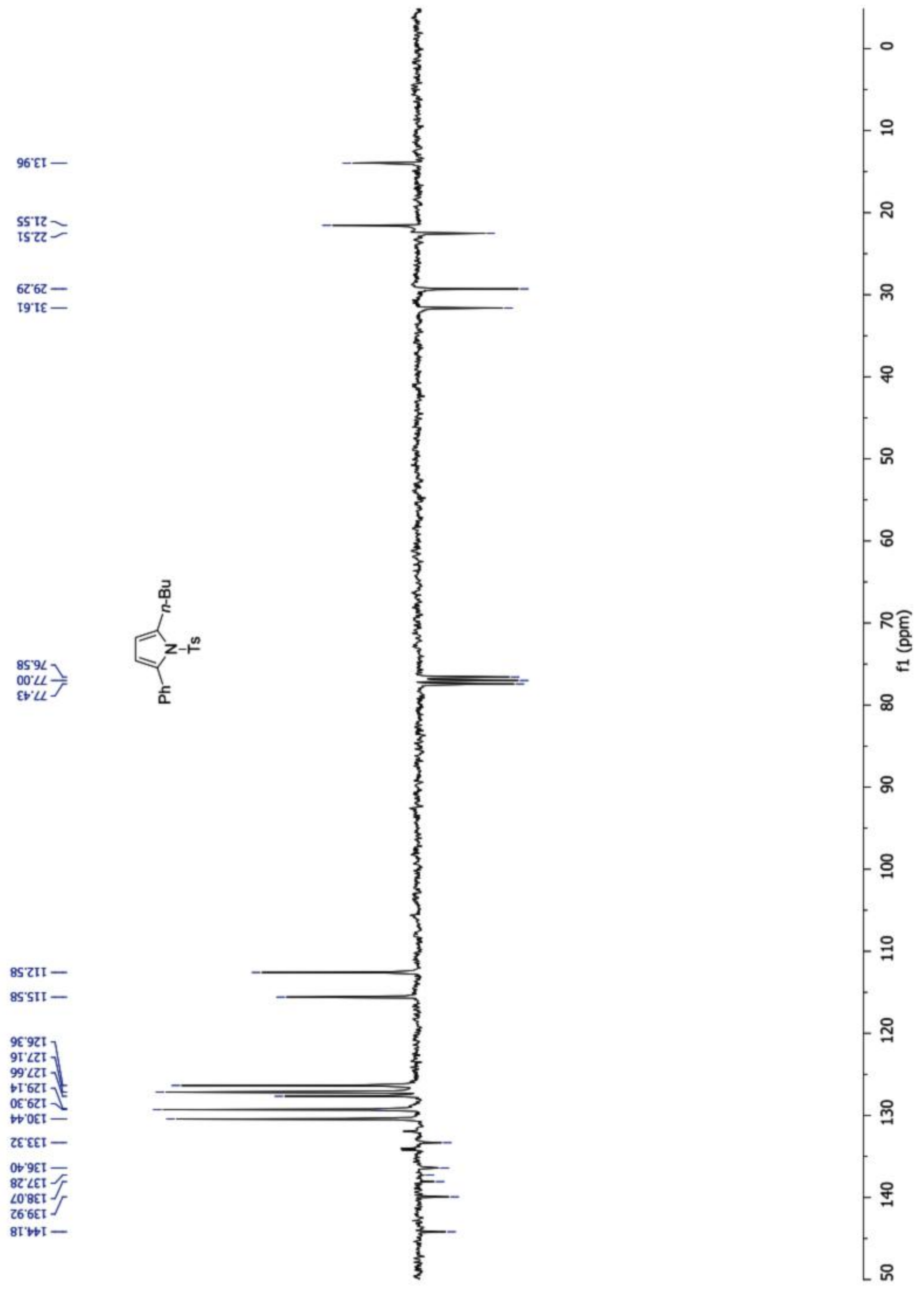

13.96
21.55
22.51
29.29
31.61
76.58
77.00
77.43
112.58
115.58
126.36
127.16
127.66
129.14
129.30
130.44
133.32
136.40
137.28
138.07
139.92
144.18
Ph
N
Ts
n-Bu
f1 (ppm)

Mixture of ^{13}C-enriched 2-butyl-4-phenyl-1-(toluene-4-sulfonyl)-1*H*-pyrroles (17a and 17b) with 2-butyl-5-phenyl-1-(toluene-4-sulfonyl)-1*H*-pyrrole (16).

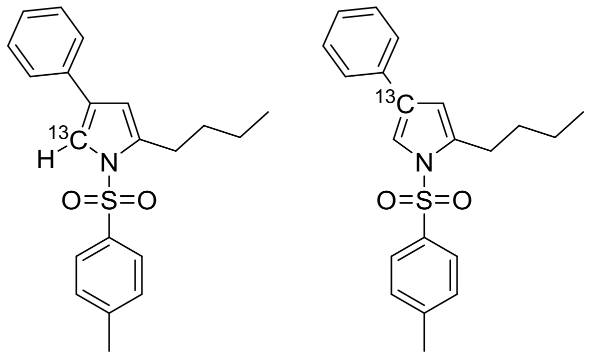

17a **17b**

Following GP5 from ^{13}C-enriched aziridine **11** (71 mg) at room temperature for 2 h. Purification by flash chromatography [hexane:ethyl acetate (25:1)] gave a mixture of 2,4-disubstituted pyrroles **17a, 17b** and 2,5-disubstituted pyrrole **16** (50 mg, 71%, **16:(17a+17b)** 1:5); HRMS *m/z* (TOF ES+) 377.1378. $C_{20}{}^{13}CH_{23}NO_2NaS$ requires 377.1381.

^{13}C-enriched 2-butyl-4-phenyl-1-(toluene-4-sulfonyl)-1*H*-pyrroles (17a/b):

δ_H (300 MHz; $CDCl_3$) 0.91 (3H, t, *J* 7.3, CH_3), 1.31–1.43 (2H, m, CH_2), 1.54–1.61 (2H, m, CH_2), 2.41 (3H, s, CH_3), 2.69 (2H, t, *J* 7.6, CH_2), 6.33 (1H, dt, *J* 1.9 and 1.0, CH), 7.29 (2H, d, *J* 8.4, 2 × CH), 7.32 (2H, d, *J* 8.4, 2 × CH), 7.37 (1H, d, *J* 8.2, CH), 7.51 (2H, dd, *J* 8.4 and 8.2, 2 × CH), 7.58 (1H, d, *J* 1.9, CH), 7.69 (2H, d, *J* 8.4, 2 × CH); δ_C (75 MHz; $CDCl_3$) 13.9 (CH_3), 21.6 (CH_3), 22.4 (CH_2), 26.9 (CH_2), 30.7 (CH_2), 110.4 (CH), 117.7 (^{13}C-enriched signal in **17a**, CH), 125.4 (2C, 2 × CH), 126.7 (2C, 2 × CH), 126.8 (CH), 126.9 (^{13}C-enriched signal in **17b** C_{quat}), 128.7 (2C, 2 × CH), 130.0 (2C, 2 × CH), 133.7 (C_{quat}), 136.3 (C_{quat}), 136.9 (C_{quat}), 144.8 (C_{quat}).

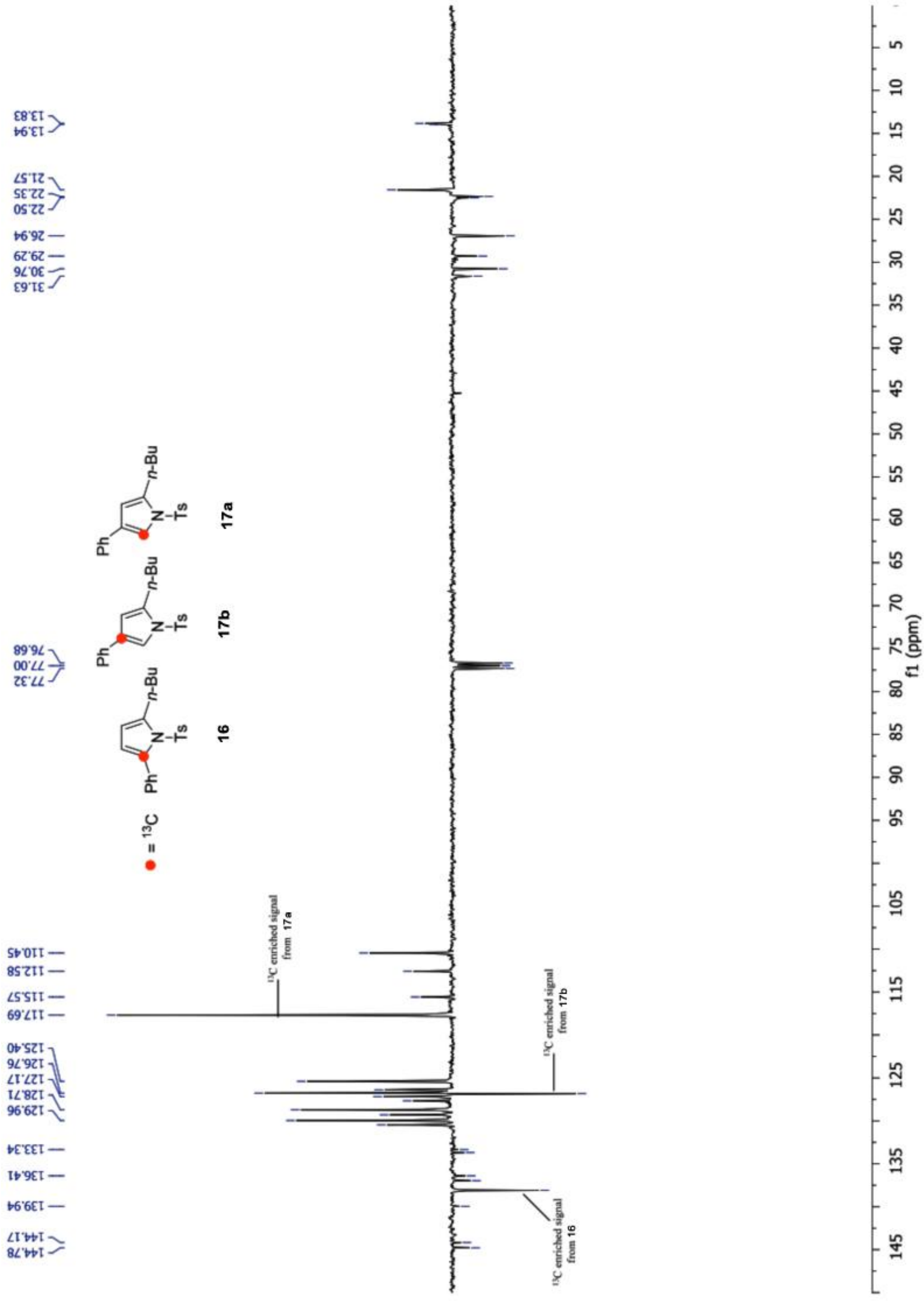
13.83
13.94
21.57
22.35
22.50
26.94
29.29
30.76
31.63
76.68
77.00
77.32
110.45
112.58
115.57
117.69
125.40
126.76
127.17
128.71
129.96
133.34
136.41
139.94
144.17
144.78
● = 13C
Ph
n-Bu
N
Ts
16
17b
17a
13C enriched signal from 17a
13C enriched signal from 17b
13C enriched signal from 16
f1 (ppm)

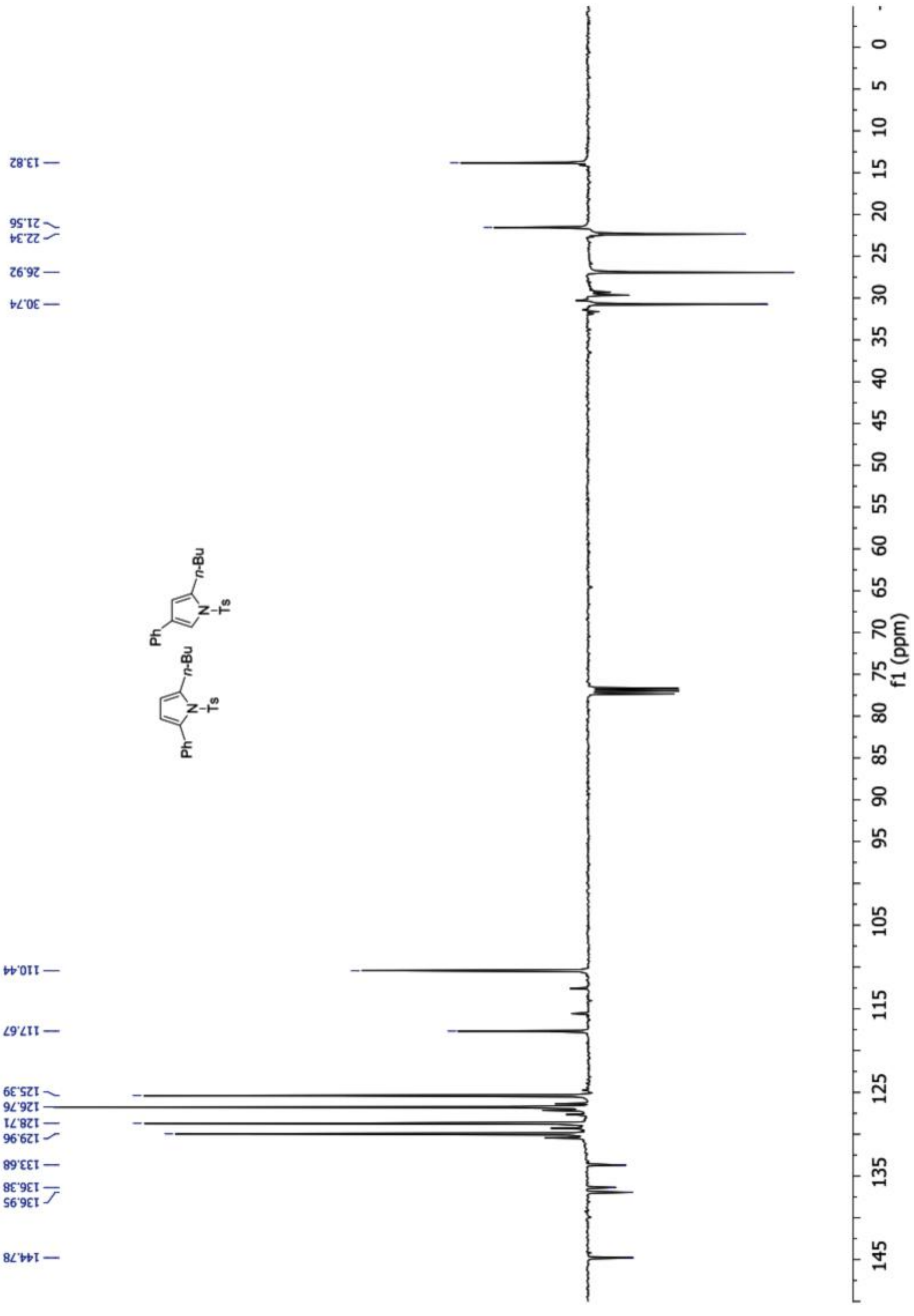

13.82
21.56
22.34
26.92
30.74
110.44
117.67
125.39
126.76
128.71
129.96
133.68
136.38
136.95
144.78
Ph
n-Bu
N
Ts
Ph
n-Bu
N
Ts
0
5
10
15
20
25
30
35
40
45
50
55
60
65
70
75
80
85
90
95
105
115
125
135
145
f1 (ppm)

Cycloisomerisation experiment using ^{13}C-labelled alkynyl aziridine 14

Mixture of ^{13}C-enriched 2,4-diphenyl-1-(toluene-4-sulfonyl)-1*H*-pyrrole (19) and 2,5-diphenyl-1-(toluene-4-sulfonyl)-1*H*-pyrrole (18)

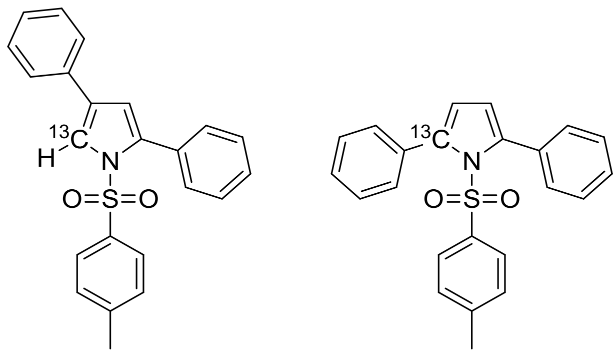

Following GP5 from ^{13}C-enriched 2-phenyl-3-(phenylethynyl)-1-(toluene-4-sulfonyl)aziridine (75 mg) at room temperature for 2 h. Purification by flash chromatography [hexane:ethyl acetate (25:1)] gave a mixture of 2,4-disubstituted pyrrole **19** and 2,5-disubstituted pyrrole **18** (49 mg, 65%, **18**:**19** 1:10); HRMS *m*/*z* (TOF ES+) 397.1075. $C_{22}{}^{13}CH_{19}NO_2NaS$ requires 397.1068.

^{13}C-enriched 2,4-diphenyl-1-(toluene-4-sulfonyl)-1*H*-pyrrole (19):

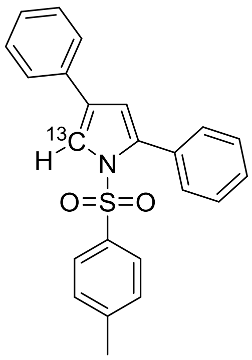

δ_H (300 MHz; $CDCl_3$) 2.34 (3H, s, CH_3), 6.49 (1H, d, *J* 2.0, CH), 7.24–7.37 (10H, m, 10 × CH), 7.53 (2H, d, *J* 7.1, 2 × CH), 7.73 (1H, d, *J* 2.0, CH); δ_C (75 MHz; $CDCl_3$) 21.6 (CH_3), 114.3 (CH), 119.5 (^{13}C-enriched signal, CH), 125.5 (2C, 2 × CH), 127.0 (CH), 127.1 (2C, 2 × CH), 127.4 (2C, 2 × CH), 128.4 (CH), 128.8 (2C, 2 × CH), 129.4 (2C, 2 × CH), 130.8 (2C, 2 × CH), 131.2 (C_{quat}), 133.3 (C_{quat}), 135.4 (C_{quat}), 136.9 (C_{quat}), 141.2 (C_{quat}), 144.7 (C_{quat}).

^{13}C-enriched 2,5- diphenyl-1-(toluene-4-sulfonyl)-1*H*-pyrrole (18):

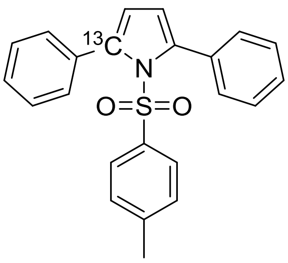

δ_H (300 MHz; $CDCl_3$) 2.34 (3H, s, CH_3), 6.24 (2H, m, 2 × CH), 7.06 (4H, m, 4 × CH), 7.35–7.46 (6H, m, 6 × CH), 7.49–7.53 (4H, m, 4 × CH); δ_C (75 MHz; $CDCl_3$) 21.6 (CH_3), 117.3 (2C, 2 × CH), 127.0 (4C, 4 × CH), 127.5 (2C, 2 × CH), 127.9 (2C, 2 × CH), 128.8 (4C, 4 × CH), 129.6 (2C, 2 × CH), 133.3 (2C, 2 × C_{quat}), 134.6 (C_{quat}), 141.3 (2C, ^{13}C-enriched signal, 2 × C_{quat}), 144.3 (C_{quat}).

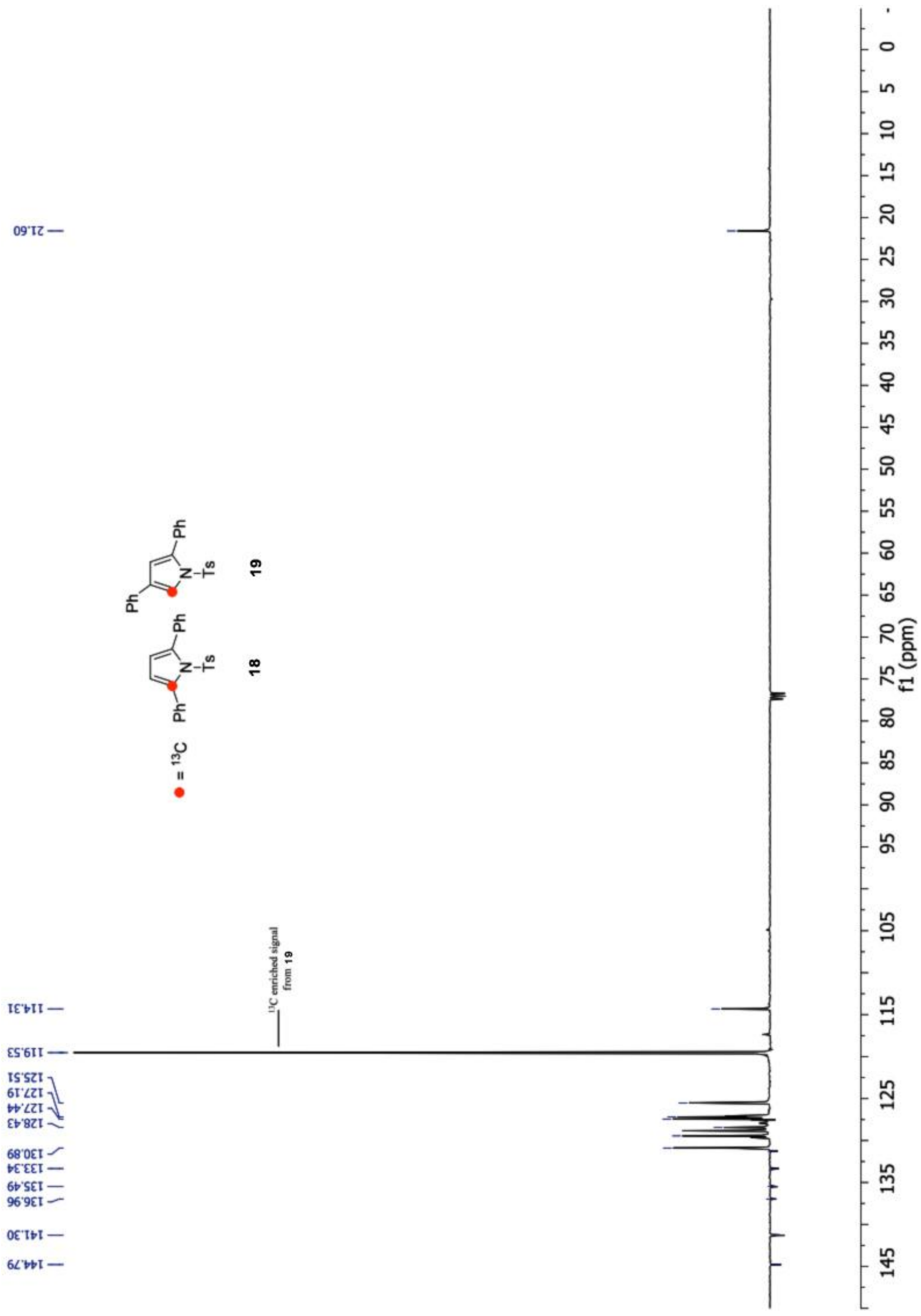

21.60
114.31
119.53
125.51
127.19
127.44
128.43
130.89
133.34
135.49
136.96
141.30
144.79
= ^{13}C
18
19
^{13}C enriched signal from 19
f1 (ppm)

Cycloisomerisation experiment using ^{13}C-labelled alkynyl aziridine 15

^{13}C-enriched 2-phenethyl-4-phenyl-1-(toluene-4-sulfonyl)-1*H*-pyrroles (21a and 21b) and 2-phenethyl-5-phenyl-1-(toluene-4-sulfonyl)-1*H*-pyrrole (20)

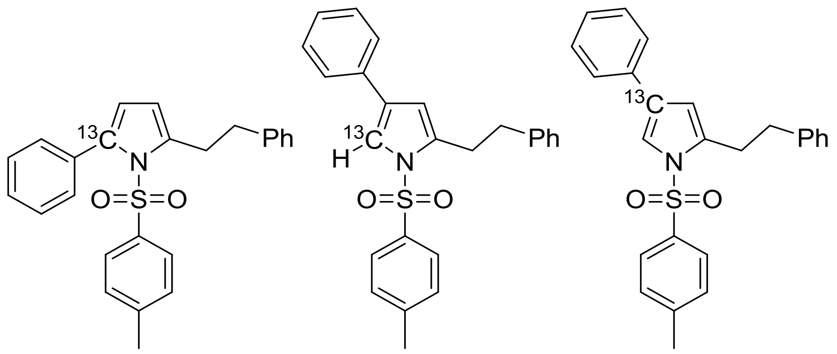

20 **21a** **21b**

Following GP5 from ^{13}C-enriched aziridine **15** (81 mg) was stirred at room temperature for 2 h. Purification by flash chromatography [hexane:ethyl acetate (20:1)] gave a mixture of 2,4-disubstituted pyrroles **21a, 21b** and 2,5-disubstituted pyrrole **20** (32 mg, 40%, **20:(21a+21b)** 1:2); HRMS *m/z* (TOF ES+) 425.1376. $C_{24}{}^{13}CH_{23}NO_2NaS$ requires 425.1381.

^{13}C-enriched 2-phenethyl-4-phenyl-1-(toluene-4-sulfonyl)-1*H*-pyrrole (21a/b):

δ_H (300 MHz; $CDCl_3$) 2.34 (3H, s, CH_3), 2.85–2.90 (2H, m, CH_2), 2.94–2.99 (2H, m, CH_2), 6.35 (1H, dt, *J* 2.0 and 0.9, CH), 7.12–7.28 (8H, m, 8 × CH), 7.30–7.33 (2H, m, 2 × CH), 7.42–7.45 (2H, m, 2 × CH), 7.54 (1H, d, *J* 2.0, CH), 7.63 (2H, d, *J* 8.4, 2 × CH); δ_C (75 MHz; $CDCl_3$) 21.6 (CH_3), 29.3 (CH_2), 35.4 (CH_2), 111.2 (CH), 118.0 (^{13}C-enriched signal for **21a**, CH), 125.5 (2C, 2 × CH), 126.1 (CH), 126.8 (2C, 2 × CH), 126.9 (CH), 127.0 (^{13}C-enriched signal for **21b**, C_{quat}), 127.3 (4C, 4 × CH), 128.4 (2C, 2 × CH), 130.1 (2C, 2 × CH), 133.6 (C_{quat}), 136.0 (C_{quat}), 138.5 (C_{quat}), 141.2 (C_{quat}), 144.9 (C_{quat}).

^{13}C-enriched 2-phenethyl-5-phenyl-1-(toluene-4-sulfonyl)-1*H*-pyrrole (20):

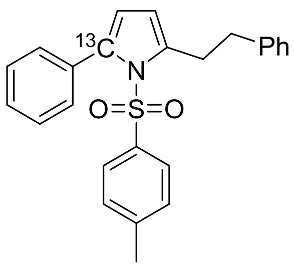

δ_H (300 MHz; $CDCl_3$) 2.36 (3H, s, CH_3), 3.05 (2H, m, CH_2), 3.24 (2H, m, CH_2), 6.02–6.09 (2H, m, 2 × CH), 7.13 (2H, d, *J* 8.0, 2 × CH), 7.19–7.31 (7H, m, 7 × CH), 7.34 (5H, 5 × CH); δ_C (75 MHz; $CDCl_3$) 21.6 (CH_3), 31.8 (CH_2), 36.3 (CH_2), 113.5 (CH), 115.7 (CH), 126.0 (CH), 126.4 (2C, 2 × CH), 127.3 (2C, 2 × CH), 127.8 (CH), 128.4 (2C, 2 × CH), 128.5 (2C, 2 × CH), 129.4 (2C, 2 × CH), 130.5 (2C, 2 × CH), 133.2 (C_{quat}), 136.3 (C_{quat}), 138.5 (^{13}C-enriched signal, C_{quat}), 138.8 (C_{quat}), 141.6 (C_{quat}), 144.4 (C_{quat}).

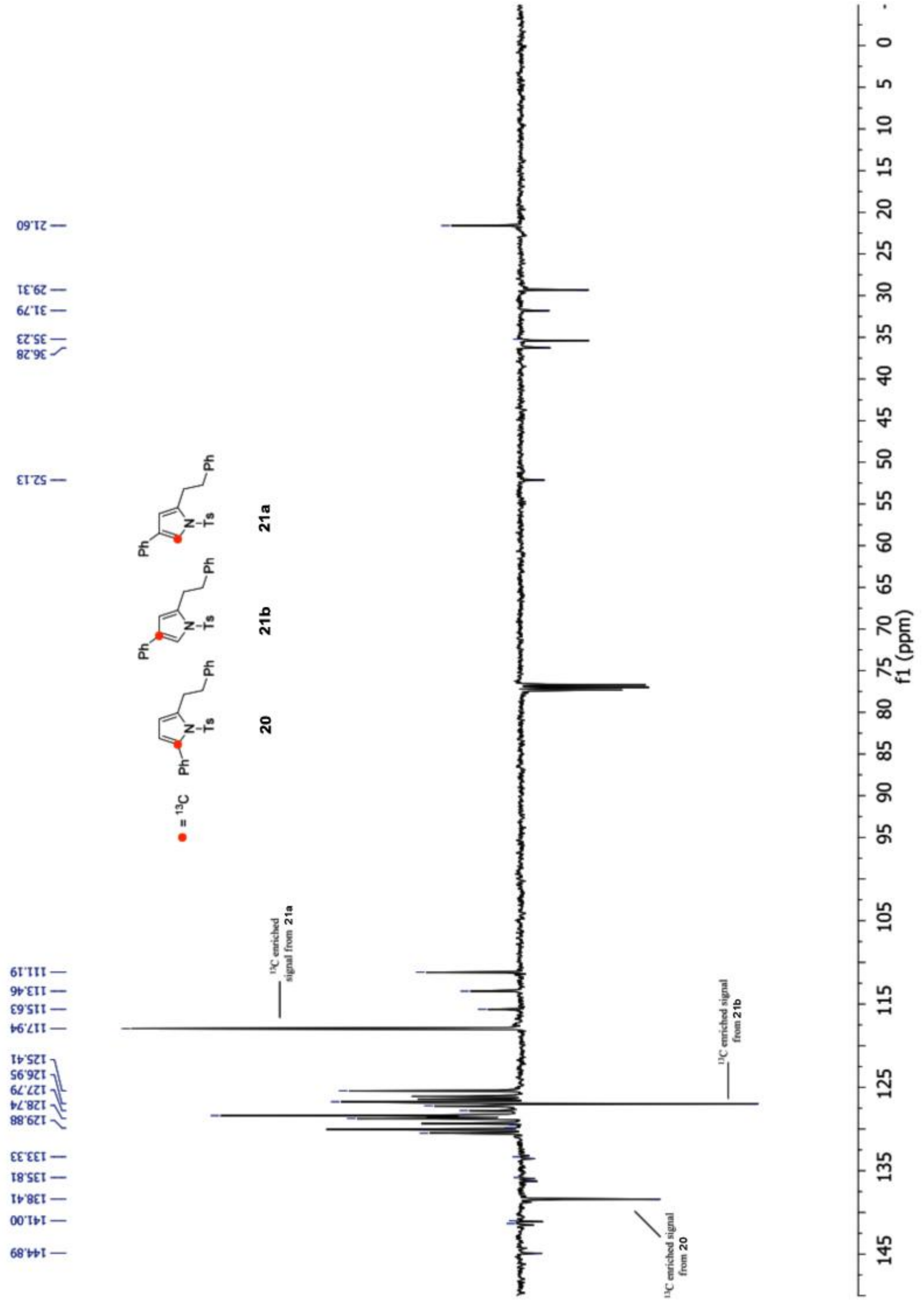
21.60
29.31
31.79
35.23
36.28
52.13
111.19
113.46
115.63
117.94
125.41
126.95
127.79
128.74
129.88
133.33
135.81
138.41
141.00
144.89
● = ^{13}C
Ph
N
Ts
Ph
20
21b
21a
^{13}C enriched signal from 21a
^{13}C enriched signal from 21b
^{13}C enriched signal from 20
f1 (ppm)

Cycloisomerisation experiments using ^{13}C-labelled alkynyl aziridine 30

^{13}C-enriched 4-phenyl-1-(toluene-4-sulfonyl)-2-(4-(trifluoromethyl)phenyl)-1*H*-pyrroles (32a) and (32b)

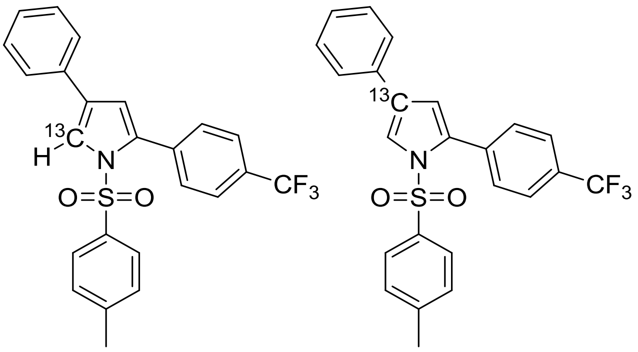

32a **32b**

Following GP5 from ^{13}C-enriched 2-phenyl-1-(toluene-4-sulfonyl)-3-((4-trifluoromethyl)phenyl)ethynyl)aziridine (88 mg) at room temperature for 2 h. Purification by flash chromatography [hexane:ethyl acetate (25:1)] gave a mixture of 2,4-disubstituted pyrroles **32a** and **32b** (39 mg, 45%); δ_H (300 MHz; $CDCl_3$) 2.36 (3H, s, CH_3), 6.55 (1H, d, *J* 1.9, CH), 7.13 (2H, d, *J* 8.1, 2 × CH), 7.26–7.31 (3H, m, 3 × CH), 7.37–7.46 (4H, m, 4 × CH), 7.51–7.55 (2H, m, 2 × CH), 7.61 (2H, d, *J* 8.1, 2 × CH), 7.76 (1H, d, *J* 1.9, CH); δ_C (75 MHz; $CDCl_3$) 21.6 (CH_3), 115.4 (CH), 120.4 (^{13}C-enriched signal in **32a**, CH), 123.6 (q, *J* 272.6, C_{quat}), 124.4 (2C, q, *J* 3.1, 2 × CH), 125.5 (2C, 2 × CH), 127.0 (2C, 2 × CH), 127.3 (CH), 128.0 (^{13}C-enriched signal in **32b**, C_{quat}), 128.9 (2C, 2 × CH), 129.6 (2C, 2 × CH), 130.2 (q, *J* 32.8, C_{quat}), 130.9 (2C, 2 × CH), 132.9 (C_{quat}), 134.9 (C_{quat}), 135.2 (C_{quat}), 135.4 (C_{quat}) 145.1 (C_{quat}); HRMS *m*/*z* (TOF ES+) 465.0939. $C_{23}{}^{13}CH_{18}NO_2F_3NaS$ requires 465.0942.

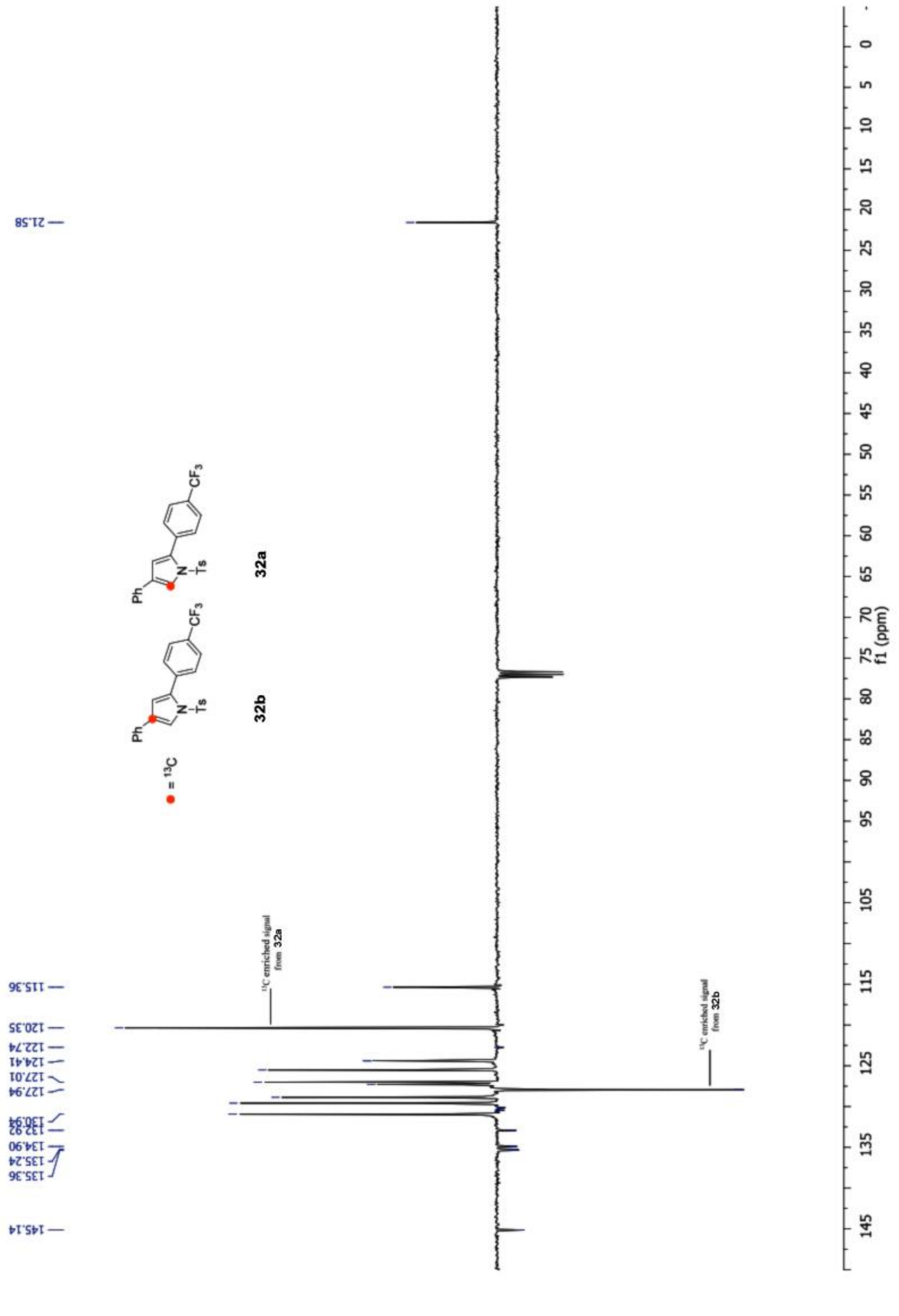

21.58
115.36
120.35
122.74
124.41
127.01
127.94
130.94
132.92
134.90
135.24
135.36
145.14
Ph
CF3
N
Ts
32a
32b
= 13C
13C enriched signal from 32a
13C enriched signal from 32b
f1 (ppm)

Cycloisomerisation experiments using ^{13}C-labelled alkynyl aziridine 31

Mixture of ^{13}C-enriched 2-(4-methoxyphenyl)-5-phenyl-1-(toluene-4-sulfonyl)-1*H*-pyrrole (33) and 2-(4-methoxyphenyl)-4-phenyl-1-(toluene-4-sulfonyl)-1*H*-pyrrole (34)

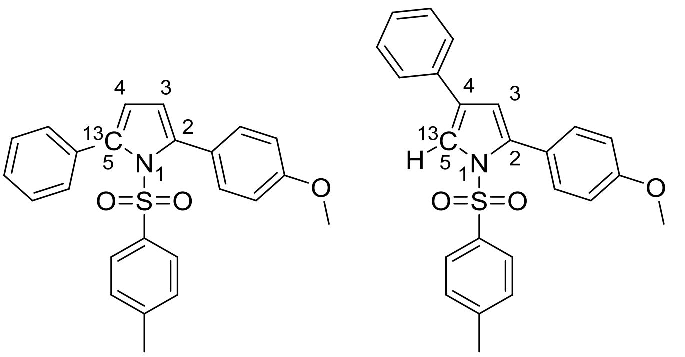

33 **34**

Following GP5 from ^{13}C enriched aziridine **31** (80 mg) at room temperature for 45 min. Purification by flash chromatography [hexane:ethyl acetate (20:1)] gave a mixture of 2,4-disubstituted pyrrole **34** and 2,5-disubstituted pyrrole **33** (8 mg, <10%, **33**:**34** 2:3).

Only a complex and a "dirty" mixture of pyrroles was obtained. Characteristic resonances of the expected 2,4 and 2,5-disubstituted pyrroles are visible in ^{1}H NMR:

2-(4-methoxyphenyl)-5-phenyl-1-(toluene-4-sulfonyl)-1*H*-pyrrole (33): δ_H (300 MHz; $CDCl_3$) 2.35 (3H, s, CH_3), 3.87 (3H, s, CH_3), 6.16 (1H, d, *J* 3.3, CH), 6.23 (1H, d, *J* 3.3, CH), 6.85–7.50 (13H, m, 13 × CH).

^{13}C NMR shows a ^{13}C-enriched signal at 140.7 ppm characteristic of ^{13}C enrichment at C-5 for a 2,5-pyrrole.

2-(4-methoxyphenyl)-4-phenyl-1-(toluene-4-sulfonyl)-1*H*-pyrrole (34): δ_H (300 MHz; $CDCl_3$) 2.35 (3H, s, CH_3), 3.88 (3H, s, CH_3), 6.43 (1H, d, *J* 2.0, CH), 6.85–7.50 (13H, m, 13 × CH), 7.70 (1H, d, *J* 2.0, CH).

^{13}C NMR shows a ^{13}C-enriched signal at 119.2 ppm characteristic of ^{13}C enrichment at C-5 for a 2,4-pyrrole.

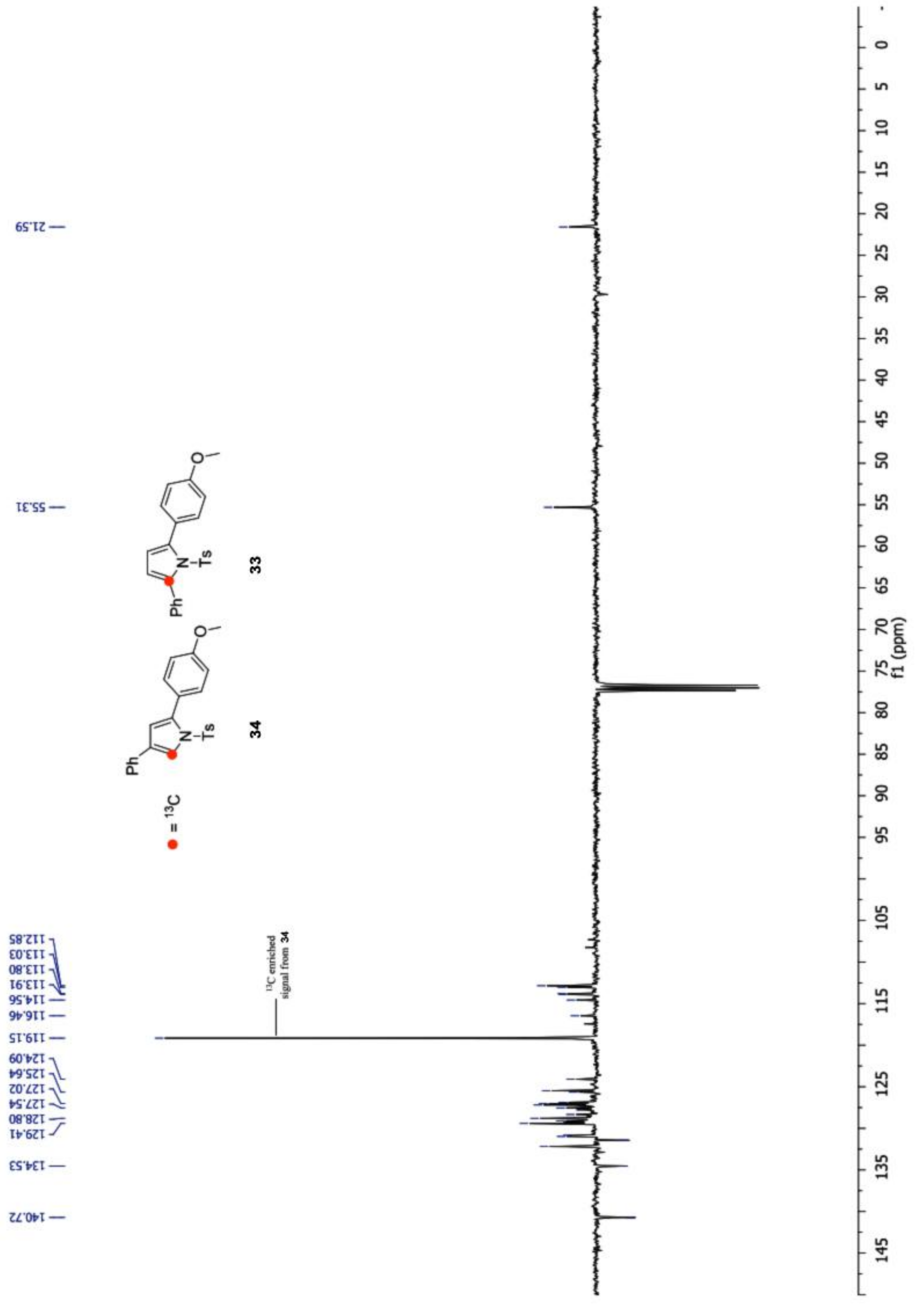

21.59
55.31
112.85
113.03
113.80
113.91
114.56
116.46
119.15
124.09
125.64
127.02
127.54
128.80
129.41
134.53
140.72
● = 13C
34
33
13C enriched signal from 34
f1 (ppm)

References

1. Janza, B.; Studer, A. *J. Org. Chem.* **2005,** *70*, 6991–6994. doi:10.1021/jo0509399
2. Dess, D. B.; Martin, J. C. *J. Am. Chem. Soc.* **1991,** *113*, 7277–7287. doi:10.1021/ja00019a027
3. Davies, P. W.; Martin, N. *J. Organomet. Chem.* **2011,** *696*, 159–164. doi:10.1016/j.jorganchem.2010.08.040
4. Trost, B. M.; Livingston, R. C. *J. Am. Chem. Soc.* **2008,** *130*, 11970–11978. doi:10.1021/ja804105m
5. Kang, S.-K.; Yoon, S.-K.; Kim, Y.-M. *Org. Lett.* **2001,** *3*, 2697–2699. doi:10.1021/ol0162825
6. Crich, D.; Karatholuvhu, M. S. *J. Org. Chem.* **2008,** *73*, 5173–5176. doi:10.1021/jo7023398
7. Yi, X.-H.; Meng, Y.; Hua, X.-G.; Li, C.-J. *J. Org. Chem.* **1998,** *63*, 7472–7480. doi:10.1021/jo9815610
